# Identification and Characterization of Odorant Binding Proteins in the Forelegs of *Adelphocoris lineolatus* (Goeze)

**DOI:** 10.3389/fphys.2017.00735

**Published:** 2017-09-26

**Authors:** Liang Sun, Qian Wang, Qi Wang, Kun Dong, Yong Xiao, Yong-Jun Zhang

**Affiliations:** ^1^Key Laboratory of Tea Quality and Safety Control, Ministry of Agriculture, Tea Research Institute, Chinese Academy of Agricultural Sciences, Hangzhou, China; ^2^State Key Laboratory for Biology of Plant Diseases and Insect Pests, Institute of Plant Protection, Chinese Academy of Agricultural Sciences, Beijing, China; ^3^Key Laboratory of Integrated Pest Management on Crops in East China, Ministry of Agriculture, Key Laboratory of Integrated Management of Crop Diseases and Pests, Ministry of Education, College of Plant Protection, Nanjing Agricultural University, Nanjing, China; ^4^College of Horticulture and Plant Protection, Yangzhou University, Yangzhou, China

**Keywords:** *Adelphocoris lineolatus*, odorant binding protein, expression profiles, phylogenetic analyses, cellular immunolocalization, gustation

## Abstract

The chemosensory system is essential for insects to detect exogenous compounds, and odorant binding proteins (OBPs) play crucial roles in odorant binding and transduction. In the alfalfa plant bug *Adelphocoris lineolatus*, an important pest of multiple crops, our understanding of the physiological roles of antenna-biased OBPs has increased dramatically, whereas OBPs related to gustation have remained mostly unexplored. In this study, we employed RNA sequencing and RACE PCR methods to identify putative OBPs from the adult forelegs of both sexes. Eight candidate OBPs were identified, and three OBPs (AlinOBP15, 16, and 17) were novel. Full-length sequence alignment and phylogenetic analyses suggested that these three candidate OBPs had characteristics typical of the insect OBP family. AlinOBP16 and 17 displayed six highly conserved cysteines, placing them in the classic OBP subfamily, whereas AlinOBP15 resembled AlinOBP14 and clustered with the Plus-C clade. Quantitative real-time PCR (qRT-PCR) revealed distinct and significant tissue- and sex-biased expression patterns. *AlinOBP15* was highly expressed in female heads, and *AlinOBP16* and *17* were strongly expressed in female antennae. In particular, *AlinOBP11*, the most abundant OBP gene in our foreleg transcriptome dataset, was predominately expressed in adult legs. Furthermore, four types of sensilla hairs were observed on the forelegs of adult *A. lineolatus*, including sensilla trichodea, setae, and two types of sensilla chaetica (Sch1 and Sch2). Anti-AlinOBP11 antiserum strongly labeled the outer sensillum lymph of Sch2, implying that it has important gustatory functions in *A. lineolatus*. Our current findings provide evidence that OBPs can be functionally expressed in the tarsal gustatory sensilla of hemipteran mirid species, broadening our understanding of OBP chemosensory function in insects and facilitating the discovery of new functional targets for the regulation of insect host-searching behaviors.

## Introduction

Host plant location is essential for phytophagous species survival and drives the rapid evolution of insect-plant interactions. Insect species encounter a wide range of environments that eventually result in different life styles and host plant adaptions (Peccoud et al., 2010). Insect foraging behaviors primarily rely on chemical sensing (Visser, 1986). During the initial step of insect host orientation, plant volatiles and the insect olfactory system play crucial roles (Takken, 1991; Li and Liberles, 2015). However, after landing on a plant, another important chemosensory repertoire, namely, gustation on tarsi and labella plays a more important role. This system enables insects to locate favorable oviposition sites, avoid plant toxins and determine whether a plant is suitable for habitation (Romani et al., 2005).

Specialized insect antennal chemosensilla, such as sensilla basiconica, house general olfactory sensory neurons (OSNs) and are responsible for recognizing host plant volatiles (Park et al., 2013; Yuvaraj et al., 2013). By contrast, gustatory chemosensilla, such as contact sensilla chaetica on tarsi, labella and wing margins, possess gustatory sensory neurons (GSNs), and express gustatory receptors (GRs), enabling insect perception of taste substances on host plant surfaces (Ave et al., 1978; Anderson and Hallberg, 1990; Isidoro et al., 2001; Leopold et al., 2003; Sun et al., 2014a). In general, chemical cues for insect host plant location, either the volatile odorants or non-volatile tastants, have poor hydrophilic characteristics, and it is often difficult for them to pass through the hydrophilic chemosensillum lymph barrier to activate odorant receptors (ORs) or GRs for chemical signal transduction. Numerous reports indicate that carrier proteins, particularly odorant binding proteins (OBPs), are highly expressed in the sensillum lymph and function as adaptor molecules between chemical cues and their receptors (Leal, 2013; Pelosi et al., 2014, 2017).

Insect OBPs are small, acidic, water-soluble proteins and were first identified in the Lepidopteran moth antennal sensillum (Vogt and Riddiford, 1981). Their homologous genes have been explored in a wide range of insect species, including moths (Gong et al., 2009; Zhang T. et al., 2011; Glaser et al., 2013; Zhang et al., 2013; Walker et al., 2016; Sun et al., 2017a), flies (Graham and Davies, 2002; Hekmat-Scafe et al., 2002; Meunier et al., 2003; Leitch et al., 2015), mosquitoes (Xu et al., 2003; Zhou et al., 2008; Pelletier and Leal, 2011; He et al., 2016), aphids (Zhou et al., 2010; Gu et al., 2013), planthopper (He and He, 2014), and bugs (Gu et al., 2011a; Ji et al., 2013; Hull et al., 2014; Yuan et al., 2015; Paula et al., 2016). Six highly conserved cysteines that form three disulfide bridges help insect OBPs fold into a large pocket for molecular uptake (Leal et al., 1999; Pelosi et al., 2013), and it is clear that OBPs in the olfactory repertoire contribute to odorant recognition (Leal, 2013; Brito et al., 2016). For instance, one subfamily of OBPs known as pheromone binding proteins (PBPs) are specifically synthesized and expressed by non-neuronal auxiliary cells (trichogen and tormogen cells) in pheromone-sensitive long trichoid sensilla. These proteins show strong binding affinities to insect sex pheromones and enhance the sensitivity and specificity of olfactory receptors to such pheromones (Wang et al., 2004; Große-Wilde et al., 2006; Sun M. et al., 2013; Chang et al., 2015; Liu et al., 2015). Suppression of PBP transcript levels can seriously disrupt the responses of male insects to female-produced sex pheromones (Dong et al., 2017). The other subfamilies of OBPs, such as general odorant binding proteins (GOBPs), have been shown to be necessary for both general odorant and insect pheromone perception (He et al., 2010; Yin et al., 2012).

The physiological functions of insect OBPs might be more complicated. In addition to the odorant detection in the olfactory system, they were also reportedly expressed in gustatory organs, including taste sensilla in labellum, tarsi, and wings and were supposed to be involved in recognition of taste compounds (Ozaki et al., 1995; Galindo and Smith, 2001; Shanbhag et al., 2001; Hull et al., 2014; Sparks et al., 2014; He et al., 2017). The study of electrophysiological responses of contact-chemoreceptor sensilla on the labellum of the blowfly, *Phormia regina* suggested that a unique type of OBP known as CRLBP could functions as a carrier for monoterpenes (Ozaki et al., 2003). Direct evidences supporting this hypothesis were reported in *Drosophila species*. For instance, two OBP genes, *Obp57d* and *Obp57e*, were co-expressed in the leg taste sensilla of *Drosophila species* and contributed to the sensation of octanoic acid and the evolution of taste perception and host-plant preference (Matsuo et al., 2007; Yasukawa et al., 2010). Suppression of *Drosophila melanogaster* feeding behavior on sweet substances by bitter compounds required OBP49a (Jeong et al., 2013). Subsequent RNAi interference assay demonstrated that OBP functions in a combinatorial and sexually dimorphic manner in the gustatory system of *D. melanogaster* (Swarup et al., 2014).

Transgenic *Bacillus thuringiensis* (Bt) cotton is commonly cultivated in China, and outbreaks of the alfalfa plant bug, *Adelphocoris lineolatus* (Goeze), and other mirid species are frequent in cotton fields (Lu et al., 2010). Furthermore, substantial evidence indicates that *A. lineolatus* can destroy many other important crops, including alfalfa (*Medicago sativa* L.), green bean (*Phaseolus vulgaris*), and tea plants (*Camellia sinensi*s; Lu and Wu, 2008). Due to the polyphagous host-feeding behavior and strong migration among different host plants (Wang et al., 2017), it is very difficult to prevent and control rapidly growing populations of mirid bugs using traditional pest management strategies. Studies of the physiological and molecular basis of insect host plant selection and adaptability could yield effective complimentary measures, particularly for species that rely heavily on chemosensing for preferential host plant searching (Koczor et al., 2012).

The molecular mechanisms of *A. lineolatus* olfaction, in particular OBP identification and their binding repertoires to plant volatiles have been extensively studied (Gu et al., 2011b; Sun L. et al., 2013; Sun et al., 2014b). Interestingly, we found that antennae-enriched or mouthpart-biased OBPs potentially bind to non-volatile plant secondary metabolites (Sun et al., 2016, 2017b). Mirid species reportedly contact the host plant surface via foreleg tarsi, and therefore, it is reasonable to hypothesize that OBPs expressed on tarsi help mirid bugs to respond to contact substances on host plant surfaces. To test this hypothesis, we first identified putative OBP genes from adult forelegs using transcriptome analysis; we then assessed tissue- and sex-biased expression patterns, with a particular focus on immunolocalization in gustatory tarsi sensilla. Screening for highly expressed OBPs in gustatory organs strongly indicates the potential for physiology functions and provides a better understanding of the molecular basis of *A. lineolatus* gustation.

## Materials and methods

### Insect rearing and tissue collection

Adult *A. lineolatus* were collected from alfalfa fields at the Langfang Experimental Station of the Chinese Academy of Agricultural Sciences, Hebei Province, China. The laboratory colony was established in plastic containers (20 × 13 × 8 cm), which were maintained at 29 ± 1°C, with 60 ± 5% relative humidity, under a 14 h light: 10 h dark cycle. Adults and newly emerged nymphs were reared on green beans and 10% honey.

For transcriptome sequencing, 300 forelegs were collected from eclosion-stage bugs of both sexes (6-d old). Various tissues from *A. lineolatus* adults of both sexes, including antennae, heads without antennae, thoraxes, abdomens, legs, and wings were collected for quantitative Real-Time PCR (qRT-PCR). Samples for each tissue were collected from three biological pools, and all specimens were immediately stored at −80°C for future use.

### cDNA library construction, transcriptome assembly, and functional annotation

Total RNA was extracted from male and female antennae using a Trizol reagent (Invitrogen, Carlsbad, CA, USA). The quantity of RNA samples was checked by using 1.1% agarose gel electrophoresis and a NanoDrop™ spectrophotometer (Thermo Scientific, Wilmington, DE, USA). The messenger RNA were further isolated from the total RNA using a PolyA (+)-tract mRNA isolation System III (Promega, Madison, WI, USA), and ~2.5 μg messenger RNA was further purified from 250 μg total RNA. The mRNAs were then sheared into ~800 nucleotides via RNA Fragmentation Solution (Autolab, Beijing, China) at 70°C for 30 s, then cleaned and condensed using an RNeasy MinElute Cleanup Kit (Qiagen, Valencia, CA, USA).

The cDNA library was generated from aforesaid obtained mRNA using the SMART cDNA Amplification Kit (Clontech, Mountain View, CA, USA) and the Ion Xpress™ Plus gDNA Fragment Library Kit (Life Technologies, Thermo Scientific, Wilmington, DE, USA), following the manufacturer's protocols. The cDNAs (300–400 bp) were purified using the Min Elute Gel Recovery Kit (Qiagen, Valencia, CA, USA) and sequenced using the Proton I chip of Ion Proton™ System (Life Technology, Thermo Scientific, Wilmington, DE, USA). Using the TagDust, LUCY, and SeqClean software programs with default parameters, short or low-quality sequences and adaptor sequences were removed (Li and Chou, 2004; Chen et al., 2007; Lassmann et al., 2009). Male and female reads were assembled separately, and all reads were assembled using the MIRA3.0 (Chevreux et al., 2004) and CAP3 software programs (Huang and Madan, 1999) with default parameters. Two steps were performed to assemble the clean reads. First, the sequence assembler Mimicking Intelligent Read Assembly MIRA3 was used with the assembly settings of a minimum sequence overlap of 30 bp and a minimum percentage overlap identity of 80%. Then, Contig Assembly Program CAP3 was used with the assembly parameters of an overlap length cutoff >30 and an overlap percent identity cutoff >90%. The resulting contigs and singletons that were more than 100 bases were retained as unigenes. BLASTX and BLASTN programs were used to perform a homology search against the GenBank non-redundant protein (nr) and nucleotide sequence (nt) databases on NCBI with an *E*-value cut-off of 1.0E-5. Gene Ontology terms were obtained from the best hits obtained from BLASTX against the nr database using the Blast2GO program (Conesa et al., 2005).

### Identification and phylogenetic analyses of putative OBPs

In addition to keyword searching, a FASTA file of non-redundant contigs was created from a local nucleotide database file using the BioEdit Sequence Alignment Editor program version 7.1.3.0, and the local TBLASTN program was performed using available bug OBPs (Table S1) as the queries (Gu et al., 2011a; Hull et al., 2014; Yuan et al., 2015). Candidate unigenes encoding putative OBPs were manually checked using the BLASTX online program at the NCBI and confirmed according to the conserved cysteine pattern feature C_1_-X_25−30_-C_2_-X_3_-C_3_-X_36−42_-C_4_-X_8−14_-C_5_-X_8_-C_6_ (Xu et al., 2009; Zhou et al., 2010).

The 5′ and 3′ regions of OBP genes were amplified using SMARTer™ RACE cDNA amplification kit (Clontech, Mountain View, CA, USA) with gene-specific primers (GSP) (Table S2). Touchdown PCR was performed as follows: 95°C for 2 min followed by 5 cycles at 94°C for 30 s, 72°C for 2 min, then 5 cycles at 94°C for 30 s, 70°C for 30 s, and 72°C for 90 s, then 30 cycles at 94°C for 30 s, 68°C for 30 s, and 72°C for 90 s, and a final 10 min incubation at 72°C. The RACE PCR products were subcloned into the pEASY-T3 vector (Transgene, Beijing, China) and sequenced. The full-length OBP genes were confirmed with LA Taq DNA polymerase (Takara, Dalian, China) by PCR using gene-specific primers (Table S2).

The full-length OBP amino acid sequence alignments were performed using the program ClustalX 2.1 with default gap penalty parameters of gap opening 10 and extension 0.2 (Thompson et al., 1997). They were then edited using the GeneDoc 2.7.0 software. The neighbor-joining tree was constructed using the program MEGA 6.0 with a p-distance model and pairwise deletion of gaps (Tamura et al., 2013). The bootstrap support for the tree branches was assessed by re-sampling amino acid positions 1,000 times.

### qRT-PCR

Total RNA for each sample was isolated using Trizol reagent (Invitrogen, Carlsbad, CA, USA). The integrity of the total RNA was examined using 1.2% agarose electrophoresis, and the purity was assessed using a NanoDrop™ instrument (Wilmington, DE, USA). First-strand cDNA was synthesized from 2 μg RNA using a FastQuant RT kit with gDNA Eraser (TianGen, Beijing, China), according to the manufacturer's instructions.

For the subsequent qRT-PCR reaction, the cDNA was diluted to a concentration of 200 ng/μL. experiments were performed using an ABI 7500 Real-Time PCR System (Applied Biosystems, Carlsbad, CA). Primers were designed using the Beacon Designer 7.90 program (PREMIER Biosoft International) and are shown in Table S2. Each reaction was performed in a total reaction volume of 25 μL, consisting of 12.5 μL of SuperReal PreMix Plus (TianGen, Beijing, China), 0.75 μL each primer (10 mM), 0.5 μL Rox Reference Dye, 1 μL sample cDNA, and 9.5 μL sterilized H_2_O. The reaction cycling parameters were as follows: 95°C for 15 min, followed by 40 cycles of 95°C for 10 s and 60°C for 32 s. The PCR products were heated to 95°C for 15 s, cooled to 60°C for 1 min, heated to 95°C for 30 s, and cooled to 60°C for 15 s to measure the dissociation curves. *A. lineolatus* ß-actin was identified as a stable reference gene between different tissue samples and was used to normalize target gene expression and correct for sample-to-sample variation (Gu et al., 2011a). For data reproducibility, the qRT-PCR reactions for each sample were performed using three technical replicates and three biological replicates. The amplification efficiencies of the target and reference gene were assessed using gradient dilution templates to examine the variation of ΔC_T_ (C_T_, _Target gene_ − C_T_, _Reference gene_) with template dilution. The absolute values of the slopes of all lines from template dilution plots (log cDNA dilution vs. ΔC_T_) were close to zero, indicating that the amplification efficiency between target genes and the reference gene was similar and the comparative 2^−ΔΔCT^ method was used to calculate relative levels between tissues (Livak and Schmittgen, 2001). Comparative analyses of target gene expression among the various tissues were performed using one-way nested analysis of variance (ANOVA), followed by Tukey's honestly significance difference (HSD) tests, using the SPSS Statistics 18.0 software program (SPSS Inc., Chicago, IL, USA).

### Scanning and transmission electron microscopy and immunocytochemical labeling

To confirm that OBPs play a role in gustatory function in the tarsi, the structures and distributions of tarsi sensilla were observed using scanning and transmission electron microscopy (SEM, TEM), and immunolocalization of AlinOBP11 on different types of tarsi sensilla were performed.

Three female and male forelegs were removed from adult *A. lineolatus*, fixed in 70% ethanol for 3 h, cleaned in an ultrasonic bath (250 W) for 10 s and finally subjected to gradient elution in an ethanol series (70, 80, 90, 95, and 100%). The samples were dried in an oven thermostat at 25°C for 10 h. After coating with gold-palladium and mounting on holders, the samples were observed using a Hitachi S570 SEM (Hitachi Ltd., Tokyo, Japan).

For TEM observation and immunocytochemical labeling, newly cut forelegs were fixed separately in a mixture of 4% paraformaldehyde and 2% glutaraldehyde in 0.1 M PBS (pH 7.4) at room temperature for at least 24 h, dehydrated in an ethanol series (30, 50, 70, 80, 90, 95, and 100%), and embedded in LR white resin (Taab, Aldermaston, Berks, UK) for polymerization at 60°C. Ultrathin sections (60–80 nm) were made using the diamond knife on a Reichert Ultracut ultramicrotome (Reichert Company, Vienna, Austria). Double-staining was performed with uranyl acetate and lead citrate, and sections were observed using a Hitachi H-7500 TEM (Hitachi Ltd., Tokyo, Japan).

The localization of AlinOBP11 on different tarsi sensilla was determined using an immunocytochemical labeling assay. A polyclonal antiserum against AlinOBP11 was produced, and its specificity was confirmed by western blotting analysis in our previous study (Sun et al., 2016). The immunocytochemical labeling assay was performed according to previously reported methods (Sun et al., 2014b). Briefly, grids that contained the ultrathin bug tarsi sections were floated in 25-μL droplets of PBSG (PBS containing 50 mM glycine) followed by droplets of PBGT (PBS containing 0.2% gelatin, 1% bovine serum albumin, and 0.02% Tween-20), and then incubated with purified rabbit anti-AlinOBP11 antiserum (dilution 1:2,000) at 4°C overnight. After washing six times with PBGT, the sections were incubated with secondary antibody (anti-rabbit IgG) coupled with 10-nm colloidal gold granules (Sigma, St. Louis, MO, USA) at a 1:20 dilution at room temperature for 90 min. Sections were subjected to optional silver intensification for 15 min, stained with 2% uranyl acetate to increase the contrast, and observed using a HITACHI H7500 TEM (Hitachi Ltd., Tokyo, Japan). Immunocytochemical assays were conducted on three biological replicates. Serum supernatant from an uninfected healthy rabbit at the same dilution rate was used as the negative control.

## Results

### RNA sequencing and *De novo* assembly

**Figure 1:**
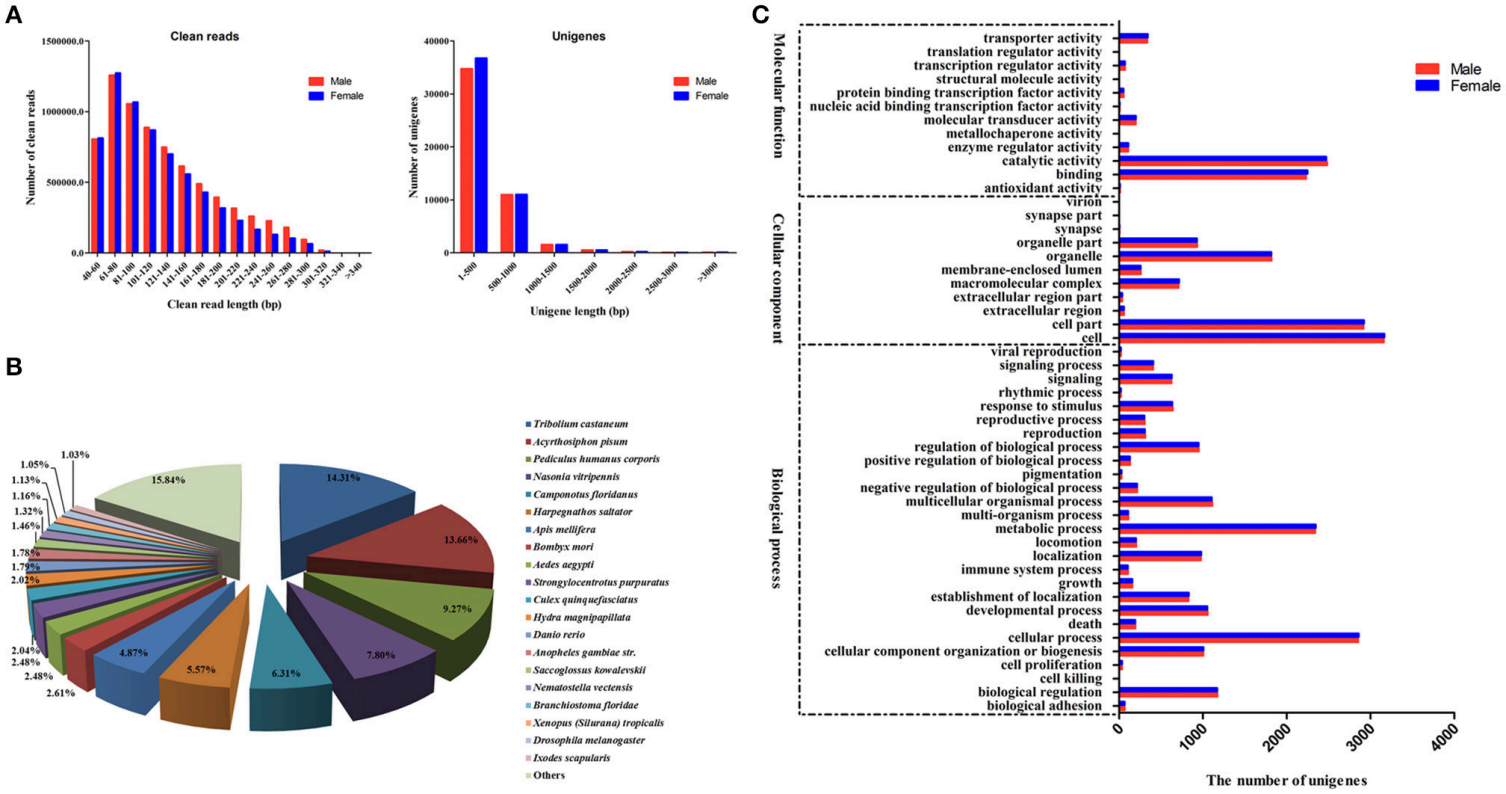
**(A)** Size distributions of the clean reads and assembled unigenes from *A. lineolatus* adult foreleg transcriptomes. **(B)** The top 20 homologous hits of the unigenes in other insect species. *A. lineolatus* unigenes were searched using BLASTX against the non-redundant protein database with a cutoff *E*-value of 10^−5^. **(C)** Gene ontology (GO) classification of unigenes according to their involvement in three functional categories: molecular function, cellular component, and biological process.

We performed RNA sequencing on male and female *A. lineolatus* adult forelegs to identify gustatory organ-biased OBPs. We obtained 7,348,393 clean reads with an average length of 127 bp for males, and 6,728,599 clean reads with an average length of 119 bp for females. High-quality fragments were assembled into 48,127 (mean length 477 bp) and 50,149 unigenes (mean length 477 bp), respectively. Subsequently, both male and female clean reads were assembled together to generate 50,801 unigenes with an average length of 469 bp (Table 1 and Figure 1A).

**Table 1:** Overview of *A. lineolatus* foreleg transcriptome sequencing and assembly process.

		**Male**	**Female**	**Total**
Clean reads	Number	7,348,393	6,728,599	14,076,992
	Average length	127	119	123
	Maximum length	434	435	435
Unigenes	Number	48,127	50,149	50,801
	Average length	477	471	469
	Maximum length	12,216	12,216	12,216

### Homology searching and functional annotation

The BLASTX program was used to annotate the acquired unigenes against an NCBI nr protein database with a cut-off *E*-value of 10^−5^. The results showed that 12,425 (24%) unigenes had BLASTX hits. The best match percentage was 14.31% for *Tribolium castaneum* sequences, followed by 13.66% for *Acyrthosiphon pisum*, 9.27% for *Pediculus humanus corporis*, 7.80% for *Nasonia vitripennis*, and 6.31% for *Camponotus floridanus* (Figure 1B). Based on the Gene Ontology (GO) annotations, 5,682 unigenes could be assigned to the following three functional categories: molecular function, cellular components and biological processes. Individual unigenes could be assigned to more than one biological process, and no significant differences were observed between sexes for each GO category. For the molecular function GO category, catalytic activity (2,482 male unigenes and 2,472 female unigenes) and binding (2,230 male unigenes and 2,249 female unigenes) were the two most abundant subcategories. For the cellular components and biological processes categories, cell (3,159 male unigenes and 3,167 female unigenes) and cellular processes (2,852 male unigenes and 2,860 female unigenes) were the most common subcategories, respectively (Figure 1C).

### Identification and full-length sequence alignments of putative OBPs

Eight candidate OBPs were identified from the *A. lineolatus* adult foreleg cDNA library by homology analysis. Five transcript-encoded OBPs, AlinOBP1, 2, 7, 11, and 14, were previously reported in *A. lineolatus* (Gu et al., 2011a). Three OBPs, which we named AlinOBP15–17, were novel, and their sequences were deposited in GenBank (accession numbers KT596720–KT596722; Table 2).

**Table 2:** BLASTX hits of putative OBPs from the *A. lineolatus* adult foreleg transcriptome.

**Gene name**	**Accession**	**Best blastx hit**				
		**Name**	**Species**	**Protein ID**	***E*-value**	**Identity (%)**
AlinOBP1	GQ477022	Odorant-binding protein 1	*Adelphocoris lineolatus*	ACZ58027	3.00E-61	100
AlinOBP2	GQ477023	Odorant-binding protein 2	*Adelphocoris lineolatus*	ACZ58028	6.00E-17	100
AlinOBP7	GQ477028	Odorant binding protein 7	*Adelphocoris lineolatus*	ACZ58085	9.00E-98	99
AlinOBP11	GQ477032	Odorant binding protein 11	*Adelphocoris lineolatus*	ACZ58082	9.00E-99	95
AlinOBP14	GQ477035	Odorant binding protein 14	*Adelphocoris lineolatus*	ACZ58086	2.00E-82	99
AlinOBP15	KT596720	Odorant-binding protein 23	*Adelphocoris suturalis*	ANA10240	1.00E-130	98
AlinOBP16	KT596721	Odorant-binding protein 21	*Apolygus lucorum*	AMQ76474	1.00E-76	93
AlinOBP17	KT596722	Odorant-binding protein 11	*Lygus lineolaris*	AHF71038	4.00E-68	83

**Figure 2:**
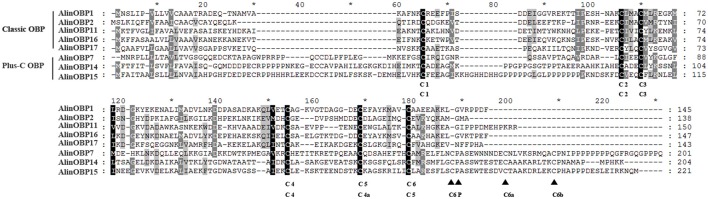
Sequence alignment of candidate OBPs identified in this study. Amino acid sequences were aligned using ClustalX 2.1 and edited using GeneDoc 2.7.0 software. Black triangles were only used to indicate conserved residues in the Plus-C sequences; conserved Cys were indicated by single letter abbreviations based on their primary sequence order.

Among the 8 identified OBPs, only one transcript, AlinOBP11, had a full-length sequence of 453 bp. As the full-length open reading frames (ORFs) of AlinOBP1, 2, and 7 were previously reported, here we report the cloned full-length sequences for the four other identified OBPs (AlinOBP14, 15, 16, and 17) based on a 5′ and 3′ RACE-PCR strategy. Full-length sequence verification showed that AlinOBP14–17 were encoded on ORFs of 615, 666, 444, and 432 bp, respectively. As shown in Figure 2, these four newly cloned OBPs can be divided into two subfamilies. AlinOBP16 and 17 have the typical six cysteine signature (C_1_-X_25−30_-C_2_-X_3_-C_3_-X_36−42_-C_4_-X_8−14_-C_5_-X_8_-C_6_) and belong to the classic OBP subfamily. In contrast, AlinOBP14 and 15 possess three extra conserved cysteines (C4a, C6a, and C6b), as well as a conserved proline (P) immediately after the sixth cysteine (C_1_-X_20−41_-C_2_-X_3_-C_3_-X_41−46_-C_4_-X_19−29_-C_4a_-X_9_-C_5_-X_8_-C_6_-P-X_9−10_-C_6a_-X_9−10_), which are typical characteristics of the insect Plus-C OBP subgroup.

### Phylogenetic analyses of OBPs

**Figure 3:**
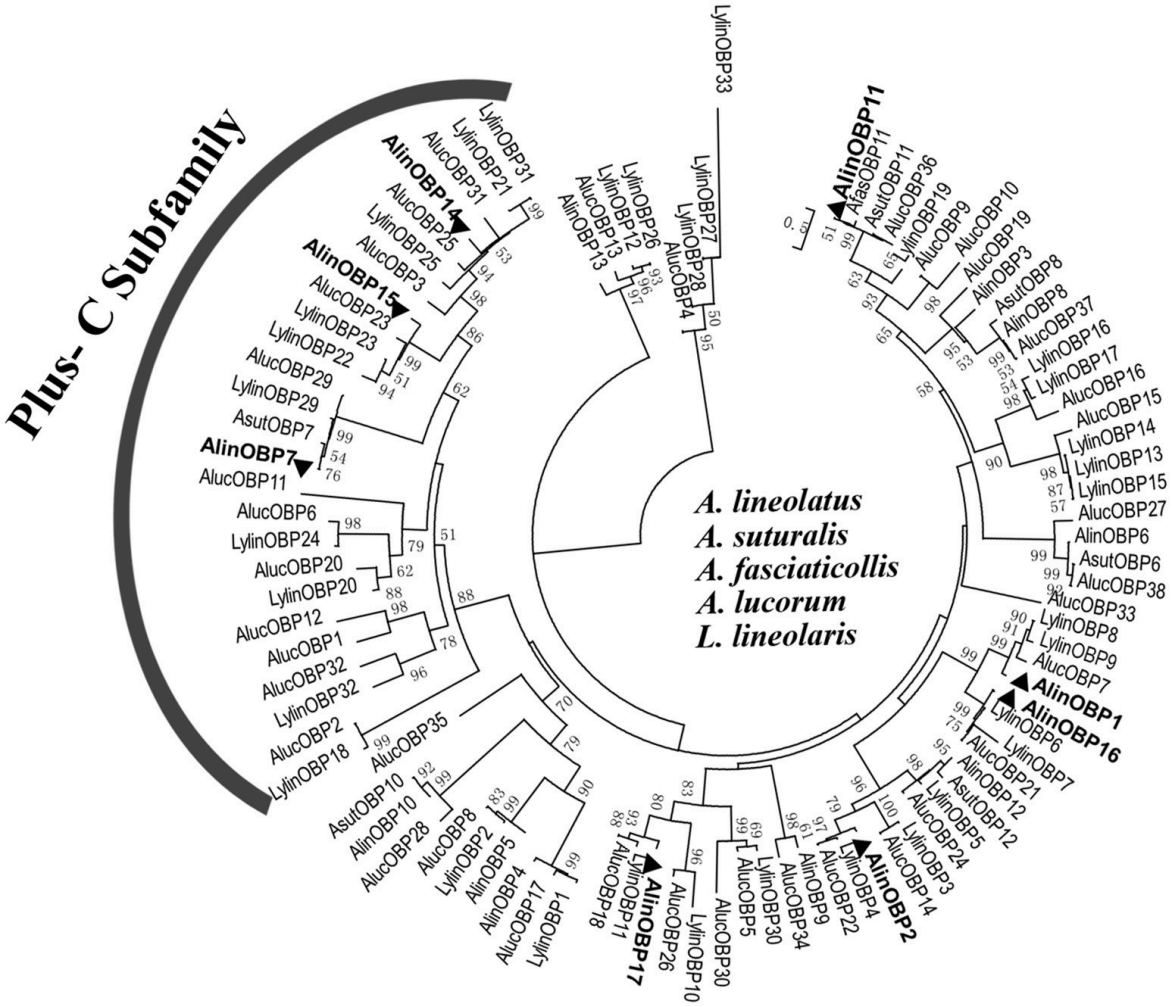
Neighbor-joining tree of candidate OBPs identified from *A. lineolatus* foreleg transcriptomes with other Hemiptera mirid bug OBPs. The tree was constructed using MEGA6.0, and values at nodes are bootstrap values based on 1,000 replicates. OBPs identified from *A. lineolatus* foreleg transcriptomes were marked in bold. OBP sequences (with signal peptides removed) used in this phylogenetic tree are shown in Table S3.

To deduce the evolutionary relationships and potential functional differences between the OBPs, 95 Hemipteran OBP sequences (Table S3) from five bug species were selected to construct phylogenetic tree (Figure 3). The phylogenetic analyses revealed that OBP within species were significantly divergent, with the amino acid identity in *A. lineolatus* only reaching 23.36%. In contrast, homologous OBPs across species shared very high similarities and clustered into the same clade with high bootstrap support, suggesting that they originated from the same ancestors and have conserved functions. Neither “minus-C” nor “dimer” OBP subfamily members were found. Only two types of motifs, referred to as “Plus-C” and “classic” OBP subgroups, were observed across the mirid bug species, and the 8 identified OBPs from *A. lineolatus* tarsi fell with these two categories. AlinOBP7, AlinOBP14, and AlinOBP15 clustered into the insect Plus-C OBP subfamily, and AlinOBP16, 17, 1, 2, and 11 and OBPs in the other bug species were assigned to the classic OBP clade.

### Tissue- and sex-biased expression patterns of candidate OBPs

**Figure 4:**
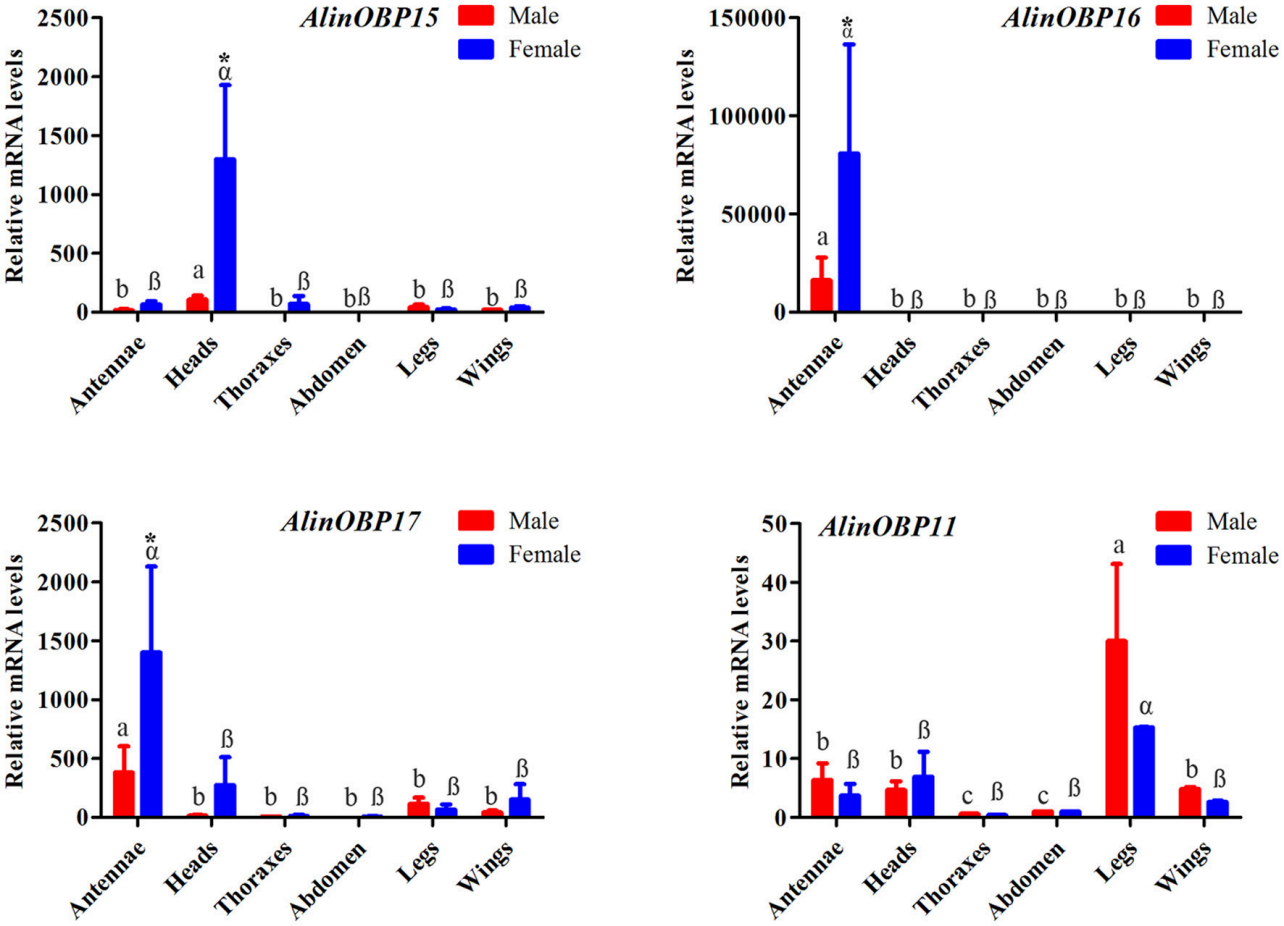
Relative transcript levels of putative OBP genes among different adult tissues of both sexes, as analyzed by qRT-PCR. Relative fold changes were normalized to transcript levels in the male abdomen. The *Alinß-actin* gene was used as reference to normalize the expression of each tested gene. Error bars represent the standard error, different letters (a, b, and c for male; a, ß represent female) above each bar denote significant differences (*P* < 0.05), and asterisks represent a significant difference between males and females (*P* < 0.05).

The tissue- and sex-biased expression profiles of the three novel OBP genes, *AlinOBP15, 16*, and *17*, were determined by qRT-PCR. AlinOBP11 was selected as a target gene to determine PCR reaction rate and reproducibility, because the RPKM value analysis revealed that AlinOBP11 was the most abundant transcript in both the male and female foreleg transcriptomes (Figure S1). This OBP gene was reported to be highly expressed in *A. lineolatus* gustatory organs legs and mouthparts (Gu et al., 2011a; Sun et al., 2016). As expected, the results of our qRT-PCR showed that *AlinOBP11* was strongly expressed in the adult legs of *A. lineolatus*, and no significant difference in expression levels was found between the sexes (Figure 4). The three novel OBP genes *AlinOBP15, 16*, and *17* shared a similar female-biased expression patterns. In particular, *AlinOBP16* and *AlinOBP17* were highly expressed in female antennae, whereas *AlinOBP15* was strongly detected in female heads (Figure 4).

### Types of sensilla on *A. lineolatus* forelegs and immunolabeling of AlinOBP11

**Figure 5:**
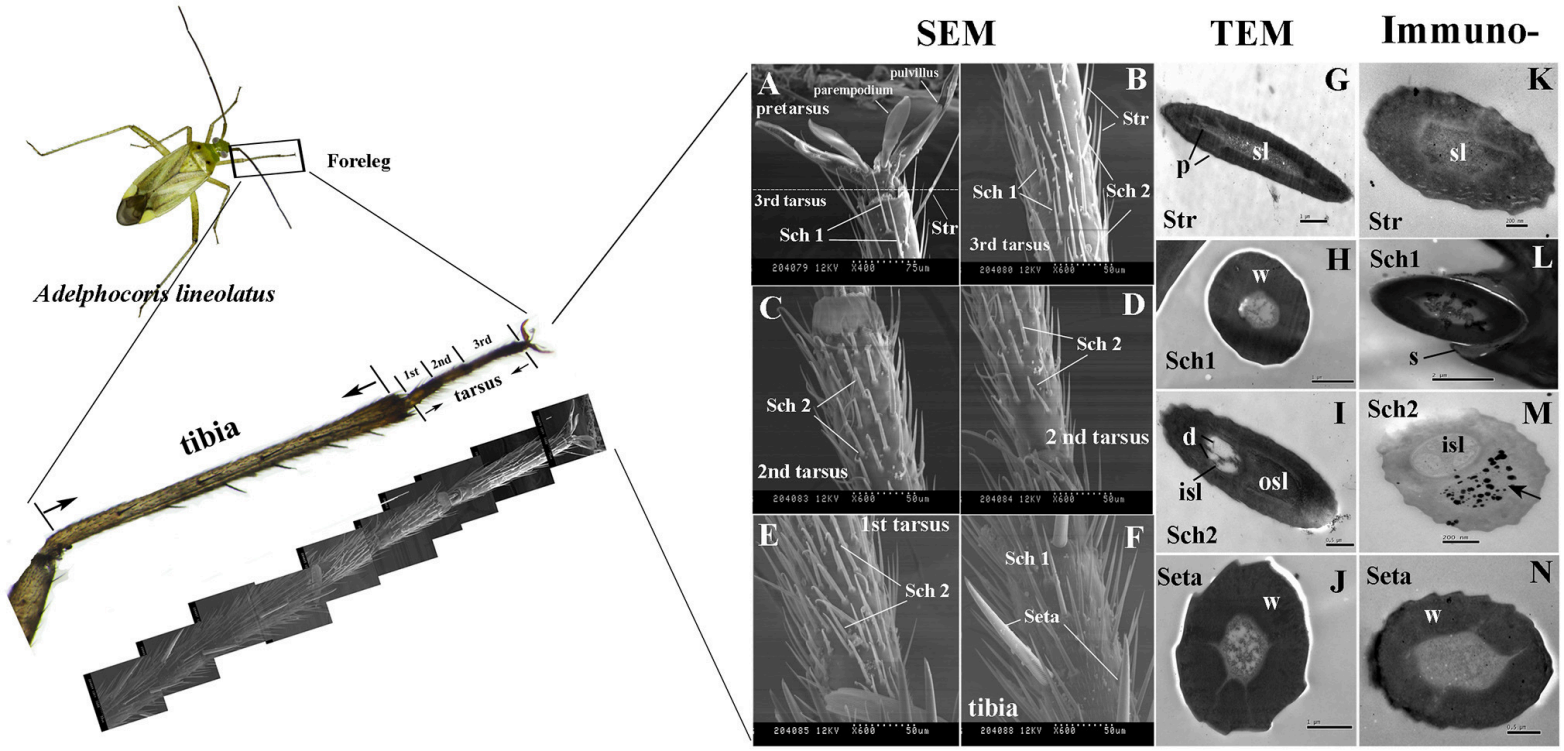
Morphology and ultrastructure of different types of sensilla present on *A. lineolatus* tarsi, and immunolabeling of AlinOBP11. Three tarsi were present on the forelegs and four different types of sensilla hairs were observed on tarsi and tibia, including sensilla trichodea (Str), setae, and two types of sensilla chaetica (Sch1 and Sch2). Strong labeling of the anti-AlinOBP11 antibody (Black spots) was detected in the outer sensillum of Sch2. The primary antibody was diluted 1–2,000, and the secondary antibody was an anti-rabbit IgG conjugated with 10-nm colloidal gold granules at a dilution of 1–20. **(A–F)** Scanning electron microscopy (SEM), **(G–J)** transmission electron microscopy (TEM), and **(K–N)** immunolocalization of AlinOBP11. Str, sensilla trichodea; Sch, sensilla chaetica; sl, sensillum lymph; isl, inner sensillum lymph; osl, outer sensillum lymph; w, sensillum wall; p, sensillum pore; d, dendrites; s, socket.

Three tarsi were found on the forelegs and four different types of sensilla hairs were present on the tarsi and tibia of adult *A. lineolatus* forelegs, including sensilla trichodea (Str), setae and two types of sensilla chaetica (Sch1 and Sch2; Figures 5A–F). Sensilla trichodea (Str) were primarily distributed on the 3rd tarsus, whereas setae were present only on the tibia. Sch1 could be found in both foreleg tarsi and tibia, and Sch2 was absent on tibia but present on all the three tarsi. Furthermore, TEM revealed that these four sensilla had distinct ultrastructures. Str had well-pore structures and one sensillum lumen. By contrast, the seta had a thick wall and no pores on the sensilla wall. SCh1 and Sch2 showed significantly different ultrastructures. Sch1 have one sensillum lumen, whereas Sch2 have two chambers and clear sensilla dendrites were found on the inner sensillum lumen rather than the outer sensillum cavity (Figures 5G–J).

We further investigated the cellular immunolocalization of AlinOBP11 because, compared with the other antennae- and head- enriched OBPs, this protein was most strongly expressed in the gustatory leg organs. Results of the immunolabeling assay showed that the anti-AlinOBP11 antibody predominately labeled the outer sensillum of Sch2, and no obvious staining was observed in either the inner sensillum lumen or the other sensilla types (Figures 5K–N).

## Discussion

In this study, we identified putative OBPs from the foreleg, an important taste organ in hemipteran insect species, and then we characterized different types of gustatory sensilla present on foreleg tarsi, where one bug OBP was predominately localized. These results provide direct morphological and molecular evidence that the foreleg tarsi of *A. lineolatus* harbor contact sensilla and that AlinOBP11, a putative carrier of bitter compounds, such as catechin and quercetin (Sun et al., 2016), plays a functional role in the tarsal gustatory repertoire.

Many reports have proposed that OBPs are expressed in gustatory organs and are involved in insect perception of hydrophobic substances to determine the host-seeking behaviors (Galindo and Smith, 2001; Matsuo et al., 2007; Jeong et al., 2013; Swarup et al., 2014). However, compared with the well-characterized process of olfactory perception, the physiological functions of OBPs associated with insect taste detection are far less clear. To date, direct evidence that insect OBPs contribute to gustation are confined to OBP28a (Swarup et al., 2014) and OBP49a (Jeong et al., 2013) as well as OBP57d/57e in *D. sechellia* (Matsuo et al., 2007). For mirid bugs, non-volatile host substances such as gossypol, catechin, and quercetin are crucial for determining whether plant species are suitable for feeding, and foreleg tarsi, which contain multiple taste sensilla, allow bugs to sensitively detect these biologically important substances. Therefore, we hypothesized that OBPs expressed on foreleg tarsi would be associated with the recognition of these contact substances on host plant surfaces. Eight candidate OBPs were identified through RNA sequencing and transcriptomic data analysis. This number was less than that previously reported for *A. lineolatus* antennae (Gu et al., 2011a) and lower than that identified in tarsi of the mosquito *Aedes aegypti* (Sparks et al., 2014). However, eight OBPs were comparable to the number found in the proboscis taste organ in the sibling species *Apolygus lucorum* (Hua et al., 2012) and the number identified in the foreleg tarsi of the swallowtail butterfly *Papilio xuthus* (Ozaki et al., 2008). Furthermore, it is likely that chemosensory genes, particularly those encode sensilla lymph-biased OBPs are differentially expressed in distinct insect tissues during specific developmental/physiology life stages and can even be induced by chemical cues (Sun et al., 2014b; Wan et al., 2015).

Insect OBPs are grouped into different subfamilies, including classic, Plus-C, Minus-C, dimer, and atypical OBPs, according to sequence variations, and these structural differences likely enable OBPs to bind to different ligands with diverse sizes and shapes (Xu et al., 2003; Zhou et al., 2004; Zhou, 2010). Among the eight candidate OBPs identified from *A. lineolatus* foreleg tarsi, five OBPs (AlinOBP1, 2, 11, 16, and 17) belong to the classic subgroup, and three OBPs (AlinOBP7, 14, and 15) have features typical of the Plus-C OBP subfamily. Phylogenetic analysis of these eight OBPs and homologous OBPs from five mirid bug species revealed that mirid OBPs can be divided into two subgroups, classic and Plus-C, and that none were related to the minus-C or other subfamily groups. Furthermore, the OBPs were generally divergent within the same species, and each bug OBP clustered with at least one OBP protein from another species; species-specific clades were not observed.

The distinct tissue-biased distributions of OBP genes in insects are strongly indicative of biological function (Hull et al., 2014). Generally, an antenna-enriched expression profile is correlated with a role in olfactory perception, whereas genes that are strongly expressed in gustatory organs, such as the proboscis, tarsi and ovipositor, could be involved in taste detection (Pelosi et al., 2014; Brito et al., 2016). Our qRT-PCR results, in combination with previous reports (Gu et al., 2011a; Sun et al., 2016), indicate that these eight OBP genes have four distinct tissue expression patterns related to distinct physiological functions. For example, *AlinOBP1, 2, 16*, and *17* were enriched in the antennae, and AlinOBP 1 and 2 were demonstrated to be physiologically important for the detection of odorants such as female bug-produced butyrate sex pheromones and host plant terpenoids (Gu et al., 2011b). The two genes that encode AlinOBP14 and 15 (two Plus-C OBPs) were strongly expressed in the head, the non-chemosensory organ and their putative ligands have not been identified. The transcript-encoded protein AlinOBP11 was highly expressed in the gustatory organs, legs, and mouthparts (Sun et al., 2016) and is therefore a good candidate for the detection of non-volatile substances.

Insect foretarsi possess gustatory receptor neurons (GRNs) that are linked to the detection of specific sweet and bitter tastants (Sanchez et al., 2014). Our cellular immunolocalization labeling indicated that the taste organ-biased AlinOBP11 is strongly expressed in the outer sensillum lymph of the contact sensilla Sch2 (Figures 5K–N). This type of sensilla is the most abundant sensilla hair present on the foretarsi of adult *A. lineolatus* (Figures 5A–F), and its ultrastructure resembles the tarsal gustatory sensilla of the honey bee *Apis mellifera* (Sanchez et al., 2014), *D. melanogaster* (Nayak and Singh, 1983), and *Helicoverpa* spp. (Zhang et al., 2010; Zhang Y. F. et al., 2011), which have been demonstrated to account for the perception of sucrose and bitter substances. Furthermore, this cellular immunolocalization is consistent with previous reports of AlinOBP11 ligand-binding, which suggests that AlinOBP11 can tightly bind the bitter substances catechin and quercetin isolated from bug host plants (Sun et al., 2016). Hence, AlinOBP11 represents an attractive target for understanding the molecular basis of gustatory coding in *A. lineolatus* foretarsi, although there is currently no direct evidence supporting that Sch2 in *A. lineolatus* responds to bitter substances such as catechin and quercetin.

To date, two OBPs in *A. lineolatus* have been implicated in the perception of bitter substances, such as catechin and quercetin. One is the antennal contact sensilla-expressed AlinOBP6 (Sun et al., 2017b), and the other is AlinOBP11, which is expressed highly in mouthparts (Sun et al., 2016) and the tarsal gustatory sensillum lymph of Sch2 (Figures 5K–N). These results indicate that mirid bug species, at least for *A. lineolatus* have evolved a complex gustatory repertoire to perceive important taste substances for host plant-seeking behavior. Such sophisticated taste recognition likely requires the activation of GRNs in taste sensilla located on antennae, mouthparts, and foretarsi and involves the cooperation of different OBPs. A combinatorial mechanism for the physiological function of OBPs in the gustatory system has been proposed in *D. melanogaster* (Swarup et al., 2014), however, this conclusion still requires *in vivo* evidence in *A. lineolatus*. In the future, gene expression modification by either RNA interference (He et al., 2011) or CRISPR/Cas9 editing (Zhu et al., 2016) should be used to clarify these issues.

## Author contributions

LS and YZ conceived and designed the experimental plan. LS, QianW, and YX preformed the experiments. LS, KD, and QiW analyzed the data. LS and QianW drafted the manuscript.

### Conflict of interest statement

The authors declare that the research was conducted in the absence of any commercial or financial relationships that could be construed as a potential conflict of interest.

## Supplementary material

Figure S1 Expression levels of putative OBP transcripts identified from the *A. lineolatus* foreleg transcriptomes assessed by RPKM values.

Table S1 The “query” sequences used in candidate OBPs identification.

Table S2 Primers used in this study.

Table S3 OBP sequences (with signal peptides removed) used in the phylogenetic tree construction.
