# Supplementary figures and images for: Identification and Characterization of Odorant Binding Proteins in the Forelegs of Adelphocoris lineolatus (Goeze)
Source: Front Physiol. 2017 Sep 26;8:735. doi: 10.3389/fphys.2017.00735 (PMC5623005; doi:10.3389/fphys.2017.00735)

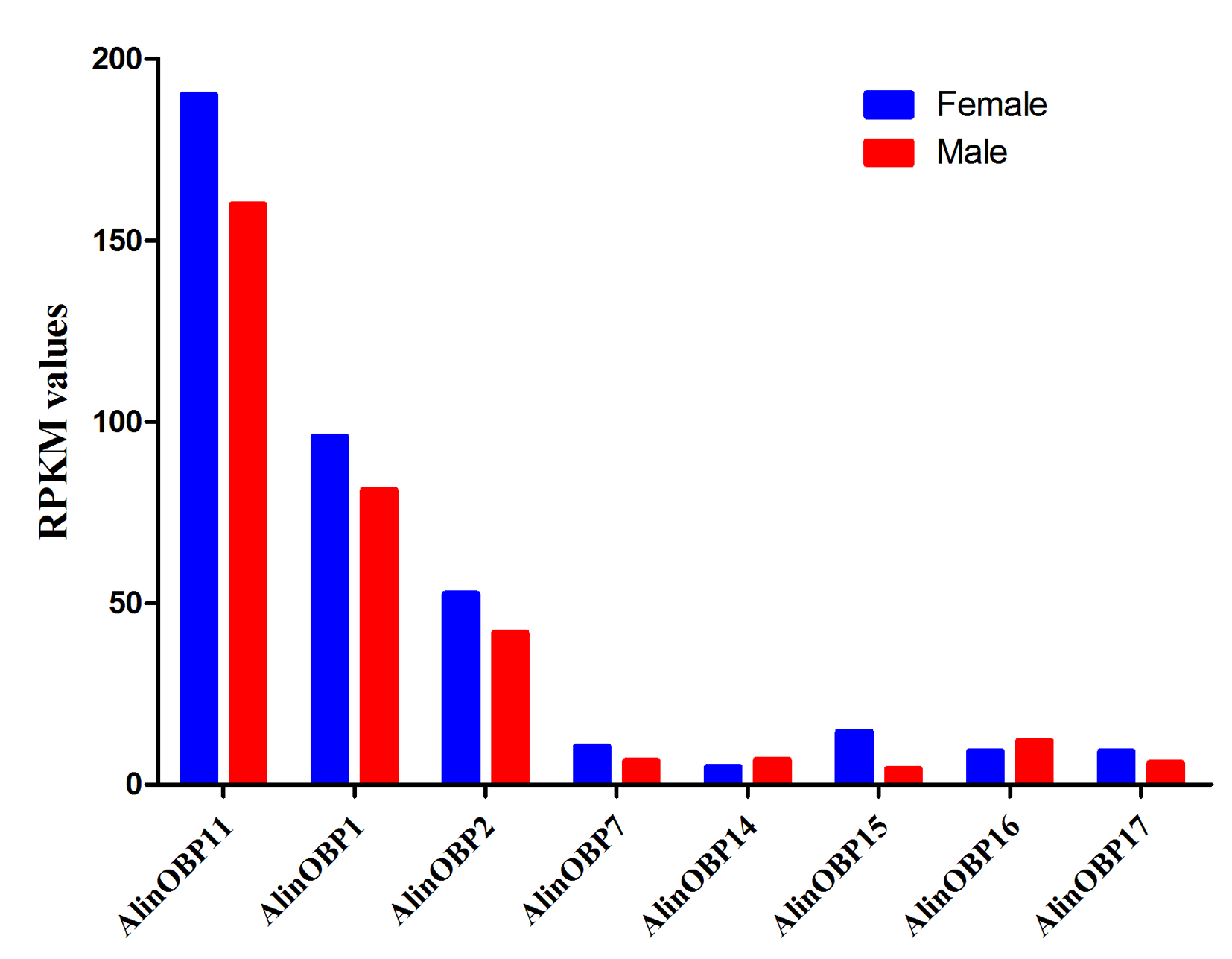

Supplement: Figure S1 — Expression levels of putative OBP transcripts identified from the A. lineolatus foreleg transcriptomes assessed by RPKM values. [file Image1.TIF]
