# Supplementary material for: Identification and Characterization of Odorant Binding Proteins in the Forelegs of Adelphocoris lineolatus (Goeze)
Source: Front Physiol. 2017 Sep 26;8:735. doi: 10.3389/fphys.2017.00735 (PMC5623005; doi:10.3389/fphys.2017.00735)
Supplement: Table S1 — The “query” sequences used in candidate OBPs identification. [file DataSheet1.DOCX]

**Table S1.** The “query” sequences used in candidate OBPs identification.

>LylinOBP1

MRILVLFTAALTCVMAGELPEEMREMAQGLHDGCVEETGVDNGLIGPCAKGNFADDQKLKCYFKCVFGNLGVISDEGELDAEAFGSILPDNMQELLPTIRGCAGTTGADPCELAMNFNKCLQKVDPVNFMVI

>LylinOBP2

MVLKMSLLLVVFVASQVLISTTEAYMSQAQMKQAMKTVRNMCIPKSGVAKEALAKMVEGEFDDSDQKLKCYLGCVLGMMQAVKNNKINLTMVRNQISKMLAPEQGQRILTAFEGCATVTGDDNCDLAFKFAKCIYDTDKELLFQAFIVP

>LylinOBP3

MAVNAKAVLFLALCGLVYVSAYQEVLKATLQDCKGGKEITQEEVDEFVKPLIPKNEEERCLMACVFRAYNVIVDGHFDPKLAYGVAKNILHENPEKLKHIKETLDYCGHEIPTKMDNECDLAGEVMACRNKYNKDHGYDQDP

>LylinOBP4

MSIKIHFFVFAAIGLACVCAYQEQLKQTIKDCQGGKEVTDDELEEFTKPLIPKNEEERCIMACVMRTYNIINNGHYDPKIAFGILKGILKDHPEKLNKIKEVMDHCGEDVPQHMDNECDLAGEIMQCEVKYQKAMGLA

>LylinOBP5

MTTKLRSVGMIVAVTIAYVCAYQEQLKATIQKCQDGREVTDDEVEEFTKPLIPKNEEERCLVACVFKEYKVIIDGHFDPVNALNVAKMVYKEYPEKWERIRDVIDHCGEDIPTHNDNECDLAGDIMKCEVKYLNSMPKITSLELLAGSIAATEEP

>LylinOBP6

MKFVLSAAVVLLVAAAVKANEKKANEKVTEIFNKCKETWPVTDEEIEQVKQKQSIPESKNVKCILACMLKEAKILRDGEYNKENAELMADVLYKDEPEHAXKSKQIIEMCSAELGTKTEGDDCEYAYKMSVCASKHAKELGVKTPEF

>LylinOBP7

VKANEKKANEKVTEIFNKCKETWPVTRRGNXTSENRSRAFPNPKNVKCILACMLKEAKILRDGEYNKENAELMADVLYKDEPEHAEKSKQIIEMCSAELGTKTEGDDCEFAFQGGQLKIMENSFTLRSLLADCLNRRYPGHVGYRYL

>LylinOBP8

MNPLIPVLLVVCAAATRGDEQTNAMVAKAFNKCHGEFPIGDDEMKGVREKSTVPDSHNAKCLMACMLKEGKILRDGKYEKENAIVMADVLNKDDPAAADKAKQLVETCATQVGSDASADECEFAYKMAVCAAGEAKKLGVRPPDF

>LylinOBP9

MNPLIPVLLVVCAAATRGDEQTNAMVAKAFNKCHGEFPIGDDEMKGVREKSTVPDSHNAKCLMACMLKEGKILRDGKYEKENAIVMADVLNKDDPAAADKAKQLVETCATQVGSDASADECEFAYKMAVCAAAWSSSTRFLKTPFLLQLCTHRSTWHTFKYEYLPGNTSSRDLTHHINIVCVT

>LylinOBP10

MTPIVAILFALLAAHVKANTKELSPVEVYKHKIHEECIKETKATPEQAKIVFNYKDVPKDDGEKCFMECVYKKSGGIDANGKYSIEGFNKLVDMKYKGEENAGAKMIVKDCSSKVAPKEGEKCSVGRTIRECLSAASKENEFFTI

>LylinOBP11

MHAAFVLIGSALLVAFVSGAPSANVKEIVQNVSKKCVAETKASPEQAKIAVSQHIPKDDVERCYLQCVYTGVGVIKDGKFSEEGGKKLVALRFHDAKEKELANKLIATCAKEIKAKDGEKCSLGRAVRECFVNHGKQVNFFPSA

>LylinOBP12

MVAECPAYSWVCQSTLSYYLHLQQTLQYIDRDPTLAMNQSSCILTLALTIFVMVVVSGFKELDSVLPQAKQEECRKESNFQGELSGDVSQNVTQELKCFAACSLVKLGLMNEKDGTINTTQLDELIAKHTAGKDAADMFKHSVVEPCLKEVNKTADYCEYSFQLVTCGMNKVKPPTTG

>LylinOBP13

MMKIAFVISVLVVLATVSAITPELDKKAKEAVAKCADVPGINEAKKEDCYAACFMTEMGYMTDGKINVENMEEANKQKWDDQQMINKGIEIDKTCAKQVGDTKGKSECAIGYDFGVCKTRLVKANCILRSILQTQLVPSPWWAHLPPLTPLKDSSTFNASFLKGVYREDMEQRNYNKTGLQPPTPLKQ

>LylinOBP14

MKIAFVVSVLVVLATVSAITPELDKKAKEAVAKCADVPEINEAKKEDCYAACFMTEMGYMTDGKINVENMEEANKQKWDDQQMINKGIEIDKTCAKQVGDTKGKSECAIGYDFGVCKTRLVKANCILRSILQXQLVPSPWWAHLPPLTPLKDSSTFNASFLKGVYRGDMEQRNYNKGKNKTGLQPPTPLKQ

>LylinOBP15

MMKIAFVTSVLVVLATVSAITPELDKKAKEAVAKCADVPGINEAKKEDCYAACFMTEMGYMTDGKINVENMEEANRQKWDDQQMINKGIEIDKTCAKQVGDTKGKSECAIGYDFGVCKTRLVKATGLQPPTPLKQ

>LylinOBP16

MKRLVFVLVTLYLLQSASGITDELRKKATEARLKCKQQVGLSDKEYQDWVKGISLPITNGGSCCEVCACWMRELGYMTDGHLNLNNMKNVNTQKWSEKANVEKANQIDTLCTARVVQDGRKECEIALDYRKCKTEMIKQNGGPPKPGST

>LylinOBP17

MKRLVFVLVTLYLLQSASGITDELRKKATETRLKCKQQVGLSDKEYQDWVKGISLPTTNGGSCCEVCACWMRELGYMTDGHLNLNNMKKRQDKYKHS

>LylinOBP18

MRSTGSECFEEVDAKLGNKTSWESDMDPYNCEKVKRMKKRHYCLHECKAKKLGVANEEGVLDFPKVKDLLLSRVNETWQKDILGQAADTCANSKFDQTWKDDTEEYKCNPQAIQFKHCVWKQVEMKCPEEHQNTGRHCKKLRSKISSETSKDSTAKETSV

>LylinOBP19

MKSFVGLIFAVALVEFASAVTKEYHDRAVAAKDKCAKEHNIKESEIQEFVKKHKLPETEDGKCMIACYMEEMKLITDGKVNVDEWKKSNKEKWDEEAHVAMADEIVDKCNEQVSPDGLAKCEYGFKLTECGLKHRLEKGLPAPNMDDVKRR

>LylinOBP20

MKLVKDDKARPPKPEGYECIDDCIMAKNGFLGTDKKIDAAKVNAAAKTSYTGEWAEPGAKMVEKCLAQVSANKEKGECTSGADIFSICMFRESFINCPEKSWTSSETCKANKERLIKCPKSIPFLNKSAK

>LylinOBP21

MLTAYMIVATLSVFFFAVALTQGQMDEDPDCRPPHPPGKEAQCCPLPDFVGVVDNFHDVMHKCSDEAGLRKPSGPPGSGTPPTAEEMAAHMSAHECADECLFKNTKYLQSNGELDKDAIKASVTKIFTGDWAALASSAADKCLASAKSEVGASAKCKSGARQMVKCFTRAMFLNCPASSWTESTECAAAKARITKCPNAMVPMIPPYPQPISANSTILDSLFACITDGEYMEFQSISSGRLG

>LylinOBP22

MPSQLPCPFFLLNIAFAHPGHFDEDPECRQPHHHRHEENDCCKVPSLFSNNKDEMHELVHKCFEEAGIKKHGPHHEHHGPPPLEDGPIPPPPPPPFSPKNDSKLDCVEQCFLKNLDLVDDEGDLKVDDLKALVTEKFSGDWASVGSSAIEKCLEKAKTEENESSKCKAGSKRVLHCHCTQHASYPKMGGSQSARKVTLDNPNPDPHDLNNVI

>LylinOBP23

MYAFTAALSFFLLNIAFAHPGHFDEDPECRQPHHHRHEENDCCKVPSLFSNNKDEMHELVHKCFEEAGIKKHGPHHEHHGPPPLEDGPIPPPPPPPFSPKNDSKFDCVEQCFLKNLDLVDDEGDLKVDDLKALVTEKFSGDWASVGSSAIEKCLEKAKTEENEPSKCKAGSKRVLHCLAREFFMNCPASDWTESEVCLAAKDRVSKCPHSLPPMHH

>LylinOBP24

MADSVGECMKLIKVKPEKGPPVPEGFDCMDTCVFSKLGFIGADNKLDPEKLAKKFSELFKGDWSALSESTLKKCLPMADVGKGVCSSGADVFKFCLIRELYMNCPASSWTKSDLCKANVERLEKCPNSLPFMNGSGLKNKSSR

>LylinOBP25

MFTTATSTIIFLFAVALTRGQMDEDPECRPPHPPGKAGDCCVQPKLFDEGDMPDVIKKCHEEAGVKRPSG

PPGSGTPPTAEEMAAHKSAHECAAECIFKNNNFIKSDGELDXDAIKATVTKMFTGDWATLASTTIDKCLA

SAKSEVDASPKCKSGADQVVRCFGRSLFIGCPASAWTESTECAAEKARLTKCPNAMPPPPHHKH

>LylinOBP26

MNQSSCILTLALTIVAMAVVSGFKELDSVLPQAKQEECRKESNFQGELSGDLSQNVTQELKCFAACSLVKLGLMNEKDGTINTTQLDELIAKHTEGKDAADMFKHSVVEPCLKEVNKTADYCEYSFQLVTCGMNKVKPPTTG

>LylinOBP27

KFYRRFKHHQIAAHLTFCVYVKTGSRDKTETPRSERPLCKAPTSAPRKLEKVINQCQEEIKYALLQEALSVLGETVSLRTALTRNRSKRETFTGEERRIAGCLLQCVYRKMKALDETGFPTATGLVKIYSEGVEDRNYYLATIQGVQQCLSRELQNRNKNPSIVKAEGYSCDVAYDVFNCVSEEIEQLCGTSP

>LylinOBP28

MEVAACLVLLAALAALTSAVDEKRPLCKAPTSAPRKLEKVINQCQEEIKYALLQEALSVLGETVSLRTALTRNRSKRETFTGEERRIAGCLLQCVYRKMKALDETGFPTATGLVKIYSEGVEDRNYYLATIQGVQQCLSRELQNRNKNPSIVKAEGYSCDVAYDVFNCVSEEIEQLCGTSP

>LylinOBP29

MNRPLLLLTAVLAVGSGQQEDCKTAPAGWPKRPPQCCDLPFPLEGMKREFGSCIRQIGNRQSSAVPTAQAVRDARLCIEECVYKGLGFMEEHNLNKDQLLEQLKKGVAGKKDWEKPMEDAVKSCHETITKRETPQEGACQDSAHEFTHCVMRQLFLSCPASEWNNNDECNLVKNRMQACPNIPPPPPPPPQGFRGQGPPQPQ

>LylinOBP30

MNSFTVLCLVASVVALTQGNPTTPNPTSSSHAASVSGGSTVSGVSKSPEEVKQKIKEQVEALTGACKSQTKITGEQAKIVATQAIPKTEAEKCFLECIYTGLQLTKDGKFNEPAARALAQKRFGNAPEDLTKANSMIDTCVKEVVVKDLNEKCALGRLIRECFVKNGAKINFFPKP

>LylinOBP31

MLTAYMIVATLSVFFFAVALTQGQMDEDPDCRPPHPPGKEAQCCPLPDFVGVVDNFHDVMHKCSDEAGLRKPSGPPGSGTPPTAEEMAAHMSAHECADECLFKNTKYLQSNGELDKDAIKASVTKIFTGDWAALASSAADKCLASAKSEVGASAKCKSGARQMVKCFTRAMFLNCPASSWTESTECAAAKARITKCPNAMVPMIPPKH

>LylinOBP32

MEVNAERNVTDEQRXAVRLCSRYTEVESGLAEAGYDCLAECFFIKLGLMGEDKTLNKENILEEVRIQFHEDXVEPARKALETCMEKKYNTKCPSGIDGTMQCFTVQLMLNCPXQNWTDGEECKETRTFMEKCGETLNYYD

>LylinOBP33

LEKVINQCQEEIKYALLQEALSVLGETVSLRTALTRNRSKRETFTGEERRIAGFEFRCDLXDGWKWSQEERGGSSPTWFEINTIPGMNTTSRSWRLSSQISSSSVFCNVYTGK

>AlinOBP13

MNISTRMISLTMAYLAAALVSGHRALDGILPQANQDECREESNFRGELNDDVGRNVTQELKCFAACSLMKLGIMNEKDGTVNMTRLDELIASHTPGKDAADVFKTTVVEPCMKEVKKSTDYCEYSYQLIACGMSKVP

>AlinOBP11

MKTFVGLIFAVALVEFASAISKEYHDKAIEAKNTCAKLHNVDDETIMTYWKNHQLPEKEPETCIVICYLKEMKLVVDGKVDADAWKASNKEKWDDEKHVAAADEIVDKCSAEVPPTENECEWGLALTKCALKHGKEAGIPPPDMEHPKRR

>AlinOBP9

MMELWKWRLALIIFGLVSCIQQTEGSQRTKQQPKSKTKESVVGATRPRDAKATECVNKVNANEEESASFFRKEIPETEAGKCLLACYLEGKGLIVGGKISSSGAARVAARAYPNNRVKTGNVKHILSHCGTIAGRESNNCEMAYKLADCTTTLSDKFRL

>AlinOBP6

MGFKFVKYRSYFFVLVIRIILCIQIKAKELTDEQKEQIFAEIKNCMESTKLTDEEFESIMAKKELPTSIEGKCFTKCLMEKMEYLEEGGKINVIAVQAGMEENMEKESEITKAKEVIQQCADSVPPEDSCEYAYGISQCMYNKMKEAGISGS

>AlinOBP4

MRIFVIFTAALTCVMAGELPEEMKEMAQGLHDSCVEETGVDNGLIAPCAKGNFADDAKLRCYFKCVFGNLGVISDEGELDAEAFGSILPDSMQELLPTIKSCGGTTGSDPCDLAMNFNKCLQKADPVNFLVI

>AlinOBP2

MSLKIQFFVFAAICAACVCAYQEQLKQTIRDCQDGKEVTDDELEEFTKPLIPRNREEKCIMACVMRTYNIISNGHYDPKIAFGILKGILKDHPEKLNKIKEVMDHCGEDVPSHMDDECDLAGEIMQCEVKYQKAMGMA

>AlinOBP12

MTTKLRSIGLVFIVSISYAFAYQELLKETIKKCQNGRDVTDDEVEEFTKPLVPKNEEERCLVACVFKEYKVIIDGHFDPVNALNVAKVVYKDYPDKVERIKDVLDHCGEDIPTHNDNECDLAGDIMKCEVKYLNSVPKMT

SLEFLAGSMAATAEP

>AlinOBP10

MFFNSVFLLVVCVSSYVTKGQELPPPGDVKNKTVVFKNSFLRSAKYCSSIYETSTLAIMALLMSEKSDDQNGKCFLNCMLQRYRLMSQDGSYNKDKFKPFLEYIPDSKFLQSIRGNLKNCISEKDPDPCEKASKFIKCFYTRARNKGEIGASKEVIPADGF

>AlinOBP8

MDTHFGLLIASLAILHTANAVINKDYLEKVVTAKDKCLKEFNVDDSVVEDFIVKYNKPQSESGKCMVACFMEERGMMKDGKTITEQVMLDNQEKWIAATHVNMGKEVIDTCDKEVPNEENDKCDLAVDYMMCLVKRGDEAGLPKMDVAQLKH

>AlinOBP5

MVLKMNLLLVVLVMSQVFFSVTEAAMSQAQMKQAMKTVRNMCIPKSGVDKEALAKMVNGEFDESDQKLKCYLGCVLGMMQAVKNNKINLTMVRNQITKMLAPERGQRILAAFESCATVTGDDNCGLAFRFAKCIYDTDKEAFIVP

>AlinOBP3

MDIRFGFIIACLAILSVANAISKEYSARMIAAKEKCQKEFNVTDSVVEDFMKRNIKPESKSGKCMVHCIMEEMGMIDDHKINTEQVKLGNKEKWDDPALVELANQVADTCDQEVFTEGRCKCLVAVEYMMCLATHGDEVGLPHVDFEDSQDS

>AlinOBP1

MNSLIPVLLVVCAAATRADEQTNAMVAKAFNKCREEFPISDDEIGGVREKTTIPESHNAKCLMACMLREGKMLRDGKYEKENALIMADVLNKDDPASADKAKQLVETCAGKVGTDAGGDECEFAYKMAVCAAEEAKKLGVRPPDF

>AlinOBP7

MNRPLLLLTAVLTVGSGQQEDCKTAPAGWPRRPPQCCDLPFPLEGMKKEFGSCIRQIGNRQSSAVPTAQAVRDARLCIEECVYKGLGFMDEHKLNKDQLLEQLKKGIADKKDWTKPMEGAVKRCHETITKRETPQEAACQDSAHEFTHCAMRELFLNCPASEWNNNDECNLVKSRMQACPNIPPPPPPPPQGFRGQGPPPQ

>AlucOBP1

MCSKYFVMLIGLTVYTSAEVINEECKDRNQSSTEYETFYNCCDLESSFNETKSKEKEEAREFCENEFEKANNVSEDEGPSPASVGQDCYFECVLKKIGAMSEDYKMDKEKVTKWFMEGSHKDFEEVGKQAMEKCYDKTYSKKYCASGVMGLLWCYSEEIVMNCPAKYWDQSEKCTAAKAYMKKCSTNPWRSED*

>AlucOBP2

MRSTGSECFEEIDAKLGNKTSLESDMDPYNCEKVKRMKKRHYCMHECKAKKLGVATEEGNLEFPKVKELLLSRVNETWQKDILGQAADTCATSKFDQTWKDDTEEYKCNPQALQFKHCVWKQVEMKCPEEHQNTGRHCKKLRSKISSETSKDIAKETSV*

>AlucOBP3

MFSSATLVCLFAVALTQGQPDEDPECRPPHPPGKDDKCCTIPELIVGENMQAMMKQCFEESGMERRPPGPPGPPGSGTPPTPEEIEAHRSAHECVDECFFKAAKFMNSDGEFDLEAMKTAAASVFTGDWAPLGSETIDECFASAKSQVSASAKCTSGAHQAKKCILRNFIINCPPSAWNDSTDCAALKARLTKCPNAMPPFPHHKH*

>AlucOBP4

MEVAACLVLLAALAALTAAVEEGRPLCKAPTTAPRKLEKVINQCQEEIKYALLQEALSVLGETVSLKTALTRNRSKRETFTGEERRIAGCLLQCVYRKMKALDETGFPTATGLVKIYSEGVEDRNYYLATIQGVQQCLSRELQSRNTNPSIVKAEGYSCDVAYDMFNCVSEQIEQLCGTSP*

>AlucOBP5

INSIIVLCLVASAVALSQGNPTTPNPSTSHVSSSAGITVSGVSKSPEEIKLKIKEQVATLTGACKTQTKLTGEQAKIVASQAIPKTEAEKCFLECIYQGLQLTKDGKFNEPAARALAQKRFGNAPEDLQKANTMIDICVKEVVVKDENEKCALGRLIRECFVKNGAKINFFPKP*

>AlucOBP6

MYDRFKLFALLALVVCCESAPPEEPAECKIPESDSAELVKCCKLNVVLDEMADSVGECMKLVKGKPEKGPPVPEGFDCMDTCVFSKLGFVANNKLDAEKLTKKFSELFKGDWSALSDSTLKKCLPMAEGAKGSCASGADVFKFCIVRELYMNCPASSWTKSDLCKANVERLEKCPHSMPFLPGTGIKKN*

>AlucOBP7

MNPLILILLVVFAAATRGEEQANALVAKAFNKCFGEFPLGDDEMKEVKDKSTVPSSHNAKCLMACMLKEGRILRGGKYELENAILMADVLNKNDHAAADKAKQLIETCAAQVGTDASADECEFAYKMALCASDEAKKLGVRPPDF*

>AlucOBP8

MVLKMKQILVVFVASQVLISTTEAVMTQAQMKQAMKTVRNMCIPKSGVDKEALAKMVEGEFDESDQKLKCYLGCVLGMMQAVKNNKINLTMVKNQISKMLAPEQGQRILAAFEGCATVTGDDNCDLAFKFAKCIYDTDKELLFQAFIVP*

>AlucOBP9

MKSFVGLIFAVALVEFASAITKEYHDRAVAAKDACLKKHPSIKESDVQEFLKKHKLPETDDGKCMIACYMEEMNLMADGKINVEEAKKTNSDKYDGEPDNKELADKLIDHCSSQVSPDGMSKCEYAYQFSKCGLEYGMKNGLTPPKMYEEQRR*

>AlucOBP10

MTYHVFFRKFDLPRISRRVRQCYYHSVPRSLSGSSRRMLEETSQHHPKRRSRVSEKHKLPETDDGECMIACYMEEKNLMADGKINVKEANQTNSDKYDGEPDNKQLAEKLIDHCSSQVSPDGMSKCEYAYQFSKCGLEYGMKNGLTPPKMYEEQRR*

>AlucOBP11

MKRVKFILVLSLLSRCSSAPTDDMAACMQITNEDSASMATCCDYVIPFSNKTMTTCDKKETSGEMSKEFECVQDCLFSSDNVLGADKKFDPVAWRKHATNTISGDWKEVIANSGSNCEGFKKVLAQSMEKKCPTSESDVSFNCMTLQWYMNCPKSAWTSSESCEASKKKLMSCFGPIFENTS*

>AlucOBP12

MTCSHFIALLSVVALSLSSGEINEECKDIENLKTQLENFYGCCDFESMIERVVRTAEEVETDRFCREERKKINSTDGKMPLASEGHDCFMECVLKRMGAMGQDFKFIREKLDDFFLRGYPEEVKQAGKLAFDKCLSKNFSKKYCASGINGLMMCLPEELVMNCPANIWSSHESCPIAKEAIKKCPSYRVMIEQE*

>AlucOBP13

MKHSSCVVPVALTIFVVAIVSGFKELDDVLPKPKQDECRKESNFQAELPSDINQNITQELKCFAACSLVKLGLMNEKDGTINMAQLEDLIAKHTGGKDAADMFKHTVVEPCMKEVNKTTDYCEYSFQLVKCGMSKVKPPSTGTEG

>AlucOBP14

MALNAKAVLLLGVCGLVYVSAYQEVLKATLKDCKGGKEITQEEVDEFMKPLIPKNEEERCLMACVFRAYNVIVDGHFDPKLAYGVAKNILHENPEKLKHIKETLDYCGHEIPTKMDNECDLAGEVMSCRNKYNIDHGYDQDP

>AlucOBP15

MMRPTAYYLFASYAALLVCVHFASVSAITPELDKRAKAAVAKCADVPRTDEAKKEDCHAGCFMSAMGYMTNGEINVKNMEEANKQKWDDQEIIKKGIQVDTTCAKQVGDTKGKSECTIGYEFSTCKKELVKKVGLPPPTPLKE

>AlucOBP16

MKRLVFVLFTLCSLQWVSGITDELKQKAQAARLTCKQQVGLSDKEFNDWVKGIALPTTDGGTCCEVCACWMRELGYLTGGRVNLENMKAVNAQKWNNLAYVELGNKIDALCSDRVLQTGRKECEIAVDFRKCKTELIQQFGGPPKPGST

>AlucOBP17

MRILVLFTAALTCVLAGELPEEMREMAQGLHDSCVGETGVDNGLIAPCAKGSFADDPKLKCYFKCVFGNLGVISDDGELDAEAFASILPDNMQALLPTIRGCGSTTGADPCDLAMNFNKCLQKADPVNFMVI

>AlucOBP18

MHAAIVLIGSALLVAYVSGAPSANVKEIVQNVSKKCAAETKASPDQAKIVLSKNIPKDDAERCFLQCVYTGVGVIKDGKFSEEGGKKLVALRFHDAKEKELANKLIATCAKEIKAKDGEKCSLGRAVRECFVNHGKQVNFFPSA

>AlucOBP19

MNSRFGIVFASLALLHITNAGNIKEGYVAKIAEIKDKCLKEHNVDHSVVEDLLKKSIKPEVKAAQCMVACFFEENGMMKDGKIVSEMVKSNNAHQYEDPADVEKANEASDMCDGEVSTDGKDKCLLAADYALCWVKRTEEAGLPQIDFANSS

>AlucOBP20

MYTFKTFFVLTLASYVIAAPPADEPAECKPMKEKEEEISKCCKLEPVTVKEQAAFVDCMKLVKDTDKKGPPKPEGFECLDDCILSKTGSLGSDKKIDPAKINAAAKTTYTGDWAEPGAKMVEKCLAQVAENKDKTVCSTSGADVYTKCIFRESYINCPEKSWTNSDACKANKERVIKCPKTLPYNAEQHKAETR

>AlucOBP21

MKFFVVSAALVLLVAAAVKANEKKANEKVTEIFNKCKETWPVTDEEIEQVKQKQSIPDSKNVKCILACMLKEAKILRDGEYNKDNAELMADVLYKDEPEHAEKSKQIIEMCSSELGTKTEGDDCEYAYKMSVCASKHAKELGVKTPEF

>AlucOBP22

MSLKIHFFVFAAIGAACVCAYQDQLKQTIKDCQGGKEVTDEELEEFTKPLIPKNEEERCIMACVMRTYNIINNGHYDPKIAFGIIKGILKDHPEKLDRIKEVMDHCGEDVPQHMDNECDLAGEIMQCEVKYQKAMGLN

>AlucOBP23

MYVFTVALSFALLNIVFTHPGHFDEDPECRPPHSHRHEEKECCKTPNLFSKNKDEMHELVHKCFEEAGIKKPHHGHHGPPPPGDEPPPPPPPPFHSKNNTKFECVEQCFLKNLELIDEEGDLKIDDFKALVGEKYTGDWASVGSAALEKCLEKTKTEEKESSKCKAGSKHVLLCIARESFINCPASDWTESEVCSDAKERVVKCPDIPPPMNH

>AlucOBP24

MSTKLRSVGMILAIAITHVCAYQEQLKETIKQCQDGREVTDDEVEEFTKPLVPKNQEERCLVACVFKEYKVIIDGHFDPVNALNVAKMVYKDYPEKWKRIKDVIDHCGEDIPTHNDNECDLAGDIMNCEVKYLNSMPKGVSLELLAGSIAATAEP

>AlucOBP25

MFTSTIFAVFLFSVALTQGQMDDDPECRPPPPPNKEGSCCTVPRLLDNADKPEVIKKCHDEAGMKRPSGPPGSGTPPTAEEMAAHKSAHECADECIFKSSNLLKSDGELDQDAIKATTTKMFTGDWSTIASTAVEKCLATAKSEVGASAKCKSGAHQMVKCFARTMFLNCPASSWTESTECAAAKTRLTKCPNAMPPPPHHSRH

>AlucOBP26

MNPTVAIIFTLLVAYVKANTKELSPSEALKQKVKVQCQQEVKATPEQLKIYDNFKDVPKDDVENCLMECMYTKTGGIGADGKYSVEGFKKLVDMKYKGEENTKARKIAADCEAKAAPKEGEKCSMGRAIRECLAAATKENEFFTI

>AlucOBP27

MARKFIKSCYTLVALLVFVGSIHVEAKELTEEQRTQLFEDLKQCKNSTDLSDDEFETIIAKKELPTSEAGKCFTKCLMEKLDIIEDAEGGKKKISVITMQASLEENMEKEDDIAKGKDIIQKCGDTVEPEDSCAYAYNISKCIYDRMKEAGISQ

>AlucOBP28

MIIEIICVLTVGISPHFIEGQELPPPGGVGNKTAVFKESFIRTAKYCSSIHETSTVAVLAILMSEESDDQNGKCFLNCMLQRYQLMSKQGAYNKDKFKPFLDYIPESRFLQSIKGNLKTCITERDPAPCEKAYKFIKCFYTRARNKDEFGKIQRK

>AlucOBP29

MNRPLLLLTAVLAVGSGQQEDCKTAPAGWPRRPPQCCDLPFPLEGMKKEFGSCIRQIGNRQSSAVPTAQAVRDARLCIEECVYKGLGFMEEHNLNKDQILQQLTKGVADKKDWTKPMEDAVKSCHETITKRETPQEGTCKDSAHEFTHCVMRQLFLSCPASEWNNNDECNLVKSRMQACPNIPPPPPPPPQGFRGQGPPPPQ

>AlucOBP30

MNAHIVLCLVASVFALSQGTPTTPTPATSRRVTVAPEDLEQAKSLRKFCTAKTGFTGITTTETSKGKDQARTATTPRPKTQLEKCYLECLYTGLQLTKDGKFNEPGARALANKRYKNAPEELRKVNSIIDFCITEVVVRDIEEMCALGRLIKECFSKYGAKNFPEL

>AlucOBP31

MFTSATFTVFLFAVTLTRGQIDEDPECRPSGPPGKEPECCTIPMKLFGDEVQEAVVKNCFDEAGMKRPSGPHGGGSPPTAEEMAAHISAHECADECVFKSGNFIKSDGGLDEDAIKAVIAKLFTGDWAPIATAAVNKCLASAKSGVSASAKCKSGAYQLSKCFQRELFLGCPASLWTESTDCSAIKARITKCPNAKVPIGHHHKH

>AlucOBP32

MSGRHSLILVLLAAVTSAEVLTDGDCPKTMPKEMKPLYKCCVVEMDSNKTISDDQKAAVDSCVNTSKSDSDANKHDCMIECIFIKLGYMGEDKTINVDYVLKEMNSLLPEDFHEQTSKSLATCMGKKFSSTECPSEIDGVMACFSTMVLMNCPAKHWTDDEECKATRKFFQKCGDSIGYRYD

>AlucOBP33

MHPWKTTCLIGMTAALMVVTAFAGLPFQNEMAVMQCKVKFDVTAEDIQLLKDSKLPSSHSGKCMMACILKKMKVMTKRGQFDLRNVQKWLRNKYQGDQANLAKGNYVAEACANTLPTLGIQDECEMAAEIMTCVRTKSKLVKKTLNGELPKEVSP

>AlucOBP34

MEHWKWRLALLIFGMVTCVPQLEGAQKSKQPSKAKTKESQVVAARPKDARAAACVTQIGPDEEEEASFYRKEIPETDKGKCLLACYLESKGVLSGGKFSSSGAAKIAARAYPNNAAKTGNVKHILSHCGTIAARETEQCQLAYRLAECTTTLADKFKL

>AlucOBP35

MINVVFVLLIGTGIVSGGFMEALIECKQQHHVSKEEAMTGESEEVKCFSECVLKKSGMMSDNNEFDEEKIQAEGARMIKNDEQKNREFEGAAKACIEKVNGENPSEKCAKGHALFKCMKEAMPMSKMRG

>AlucOBP36

MKTFVGLIFAVALVEFASAVSKEYHDKAIAAKNTCAKLHNVDDETIMKFWKAHQLPEKEPETCIIICYMKEMKLVVDGKVDADAWKASNKEKWDDEKHVAAADEIVDKCSAEVPPTENECEWGLALTKCALKHGKEAGIPPPDMEHPKRR

>AlucOBP37

MDTHFGLLIASLAILHTANAVINKDYLEKVVTAKDKCLKEFNVDDSVVEDFIVRYNKPQSESGKCMVACYMEERGMMKDGKTITEQVMLDNQEKWIAATHVNMGKEVIDTCDKEVPNEKNDKCDLAVDYMMCLVKRGDEAGLPKMDVAQLKH

>AlucOBP38

MGFKFVKYRSYFFVLVIHIILCIQIKAKELTDEQKEQIFAEIKNCMESTKLTDEEFESIMAKKELPTSKEGKCFTKCLMEKMEYLEEGGKINVIAVQAGLEENMEKESEITKAKEIIQQCADTVPPEDSCEYAYGISQCMYTKMKEAGISGGP

**Table S2.** Primers used in this study.

| **Gene clone** | **Sequence (5′-3′)** |
| --- | --- |
| AlinOBP14 5’-Race PCR GSP | TGATGGCGTCCTTGTCCAGTTCTCC |
| *AlinOBP15* 5’-Race PCR GSP | GGAGCCAGCTTTGCACTTTGTCG |
| *AlinOBP15* 3’-Race PCR GSP | TTCACCGGAGACTGGGCGTCAG |
| *AlinOBP16* 5’-Race PCR GSP | GCTTGCTCTTCTCAGCGTGTTCAGG |
| *AlinOBP17* 5’-Race PCR GSP | CGTGGAACCTCATGGCGACCAACTT |
| **qPCR** |  |
| *AlinOBP11*-sense | CATTGTTATCTGCTACTTG |
| *AlinOBP11*-Anti-sense | TTTCATCATCCCATTTCT |
| *AlinOBP15*-sense | GAAGAAGGAGAGGTCAA |
| *AlinOBP15*-Anti-sense | CCAGGCAGATAAGGATT |
| *AlinOBP16*-sense | GAGCAAGCAAATCAT |
| *AlinOBP16*-Anti-sense | GTATTCGCAGTCATC |
| *AlinOBP17*-sense | AAGAAGGAGGAAATAAGTTG |
| *AlinOBP17*-Anti-sense | CAGGTGTTGATAAGTTGTT |
| *β-actin*-Sense | GAAACCTTCAACACACCCGC |
| *β-actin*-Anti-sense | GGTAGTCGGTCAAGTCACGG |

**Table S3.** OBP sequences (with signal peptides removed) used in the phylogenetic tree construction.

>AlinOBP1

DEQTNAMVAKAFNKCREEFPISDDEIGGVREKTTIPESHNAKCLMACMLREGKMLRDGKYEKENALIMADVLNKDDPASADKAKQLVETCAGKVGTDAGGDECEFAYKMAVCAAEEAKKLGVRPPDF

>AlinOBP2

YQEQLKQTIRDCQDGKEVTDDELEEFTKPLIPRNREEKCIMACVMRTYNIISNGHYDPKIAFGILKGILKDHPEKLNKIKEVMDHCGEDVPSHMDDECDLAGEIMQCEVKYQKAMGMA

>AlinOBP3

ISKEYSARMIAAKEKCQKEFNVTDSVVEDFMKRNIKPESKSGKCMVHCIMEEMGMIDDHKINTEQVKLGNKEKWDDPALVELANQVADTCDQEVFTEGRCKCLVAVEYMMCLATHGDEVGLPHVDFEDSQDS

>AlinOBP4

GELPEEMKEMAQGLHDSCVEETGVDNGLIAPCAKGNFADDAKLRCYFKCVFGNLGVISDEGELDAEAFGSILPDSMQELLPTIKSCGGTTGSDPCDLAMNFNKCLQKADPVNFLVI

>AlinOBP5

AMSQAQMKQAMKTVRNMCIPKSGVDKEALAKMVNGEFDESDQKLKCYLGCVLGMMQAVKNNKINLTMVRNQITKMLAPERGQRILAAFESCATVTGDDNCGLAFRFAKCIYDTDKEAFIVP

>AlinOBP6

KELTDEQKEQIFAEIKNCMESTKLTDEEFESIMAKKELPTSIEGKCFTKCLMEKMEYLEEGGKINVIAVQAGMEENMEKESEITKAKEVIQQCADSVPPEDSCEYAYGISQCMYNKMKEAGISGS

>AlinOBP7

QQEDCKTAPAGWPRRPPQCCDLPFPLEGMKKEFGSCIRQIGNRQSSAVPTAQAVRDARLCIEECVYKGLGFMDEHKLNKDQLLEQLKKGIADKKDWTKPMEGAVKRCHETITKRETPQEAACQDSAHEFTHCAMRELFLNCPASEWNNNDECNLVKSRMQACPNIPPPPPPPPQGFRGQGPPPQ

>AlinOBP8

VINKDYLEKVVTAKDKCLKEFNVDDSVVEDFIVKYNKPQSESGKCMVACFMEERGMMKDGKTITEQVMLDNQEKWIAATHVNMGKEVIDTCDKEVPNEENDKCDLAVDYMMCLVKRGDEAGLPKMDVAQLKH

>AlinOBP9

SQRTKQQPKSKTKESVVGATRPRDAKATECVNKVNANEEESASFFRKEIPETEAGKCLLACYLEGKGLIVGGKISSSGAARVAARAYPNNRVKTGNVKHILSHCGTIAGRESNNCEMAYKLADCTTTLSDKFRL

>AlinOBP10

QELPPPGDVKNKTVVFKNSFLRSAKYCSSIYETSTLAIMALLMSEKSDDQNGKCFLNCMLQRYRLMSQDGSYNKDKFKPFLEYIPDSKFLQSIRGNLKNCISEKDPDPCEKASKFIKCFYTRARNKGEIGASKEVIPADGF

>AlinOBP11

ISKEYHDKAIEAKNTCAKLHNVDDETIMTYWKNHQLPEKEPETCIVICYLKEMKLVVDGKVDADAWKASNKEKWDDEKHVAAADEIVDKCSAEVPPTENECEWGLALTKCALKHGKEAGIPPPDMEHPKRR

>AlinOBP12

MTTKLRSIGLVFIVSISYAFAYQELLKETIKKCQNGRDVTDDEVEEFTKPLVPKNEEERCLVACVFKEYKVIIDGHFDPVNALNVAKVVYKDYPDKVERIKDVLDHCGEDIPTHNDNECDLAGDIMKCEVKYLNSVPKMTSLEFLAGSMAATAEP

>AlinOBP13

LVSGHRALDGILPQANQDECREESNFRGELNDDVGRNVTQELKCFAACSLMKLGIMNEKDGTVNMTRLDELIASHTPGKDAADVFKTTVVEPCMKEVKKSTDYCEYSYQLIACGMSKVP

>AlinOBP14

QMDDDPECRPPPPPNKEGECCAVPAHILEGKGKDIHEIMKTCADEAGMKPPGPPGSGTPPTAEERAAHKIAHECADECLYKSSNLLTSAGELDKDAIKALVTKLYTGDWATAATTAIDKCLASAKGEVEATSKCKSGSFQLSRCFMRSMFLGCPASSWTESTECAAAKARLTKCPNAMAPMPHKK*

>AlinOBP15

HPGHFDEDPECRPPHHHRLEEKDCCKIPNLFSKSKDEMHELVHKCFEEAGIKKHGHHDHHGPPPPPGLGLPPPPPPPPKNDSKFDCVEQCFLKNLELINEEGEVKVDELKALIAEKFTGDWASVGSSAIEKCLEKSKTEENDSTKCKAGSKRILICLARESFLSCPASEWTESDVCTAAKDRLEKCPHAPPPDESLEIRKNQM*

>AlinOBP16

NEKKANEKVTEIFNKCKETWPVTDEEIEQVKQKNSIPESKNVKCILACMLKEAKVLKDGEYNKDNAELMADVLYKDEPEHAEKSKQIIELCSAELGTKTDGDDCEYAYKMSVCAAKHAKELGVKTPEF*

>AlinOBP17

APPSVKEIVQNVSKKCAAETKASPEQAKIILTQNIPKNDVERCYLQCVYSGVGVIKDGKFSQEGGNKLVAMRFHDAKEKELAKQLINTCAKEIKAKDGEKCSLGKGIRQCFVAHGKEVNFFPHA*

>LylinOBP1

GELPEEMREMAQGLHDGCVEETGVDNGLIGPCAKGNFADDQKLKCYFKCVFGNLGVISDEGELDAEAFGSILPDNMQELLPTIRGCAGTTGADPCELAMNFNKCLQKVDPVNFMVI

>LylinOBP2

YMSQAQMKQAMKTVRNMCIPKSGVAKEALAKMVEGEFDDSDQKLKCYLGCVLGMMQAVKNNKINLTMVRNQISKMLAPEQGQRILTAFEGCATVTGDDNCDLAFKFAKCIYDTDKELLFQAFIVP

>LylinOBP3

YQEVLKATLQDCKGGKEITQEEVDEFVKPLIPKNEEERCLMACVFRAYNVIVDGHFDPKLAYGVAKNILHENPEKLKHIKETLDYCGHEIPTKMDNECDLAGEVMACRNKYNKDHGYDQDP

>LylinOBP4

YQEQLKQTIKDCQGGKEVTDDELEEFTKPLIPKNEEERCIMACVMRTYNIINNGHYDPKIAFGILKGILKDHPEKLNKIKEVMDHCGEDVPQHMDNECDLAGEIMQCEVKYQKAMGLA

>LylinOBP5

YQEQLKATIQKCQDGREVTDDEVEEFTKPLIPKNEEERCLVACVFKEYKVIIDGHFDPVNALNVAKMVYKEYPEKWERIRDVIDHCGEDIPTHNDNECDLAGDIMKCEVKYLNSMPKITSLELLAGSIAATEEP

>LylinOBP6

NEKKANEKVTEIFNKCKETWPVTDEEIEQVKQKQSIPESKNVKCILACMLKEAKILRDGEYNKENAELMADVLYKDEPEHAXKSKQIIEMCSAELGTKTEGDDCEYAYKMSVCASKHAKELGVKTPEF

>LylinOBP7

VKANEKKANEKVTEIFNKCKETWPVTRRGNXTSENRSRAFPNPKNVKCILACMLKEAKILRDGEYNKENAELMADVLYKDEPEHAEKSKQIIEMCSAELGTKTEGDDCEFAFQGGQLKIMENSFTLRSLLADCLNRRYPGHVGYRYL

>LylinOBP8

DEQTNAMVAKAFNKCHGEFPIGDDEMKGVREKSTVPDSHNAKCLMACMLKEGKILRDGKYEKENAIVMADVLNKDDPAAADKAKQLVETCATQVGSDASADECEFAYKMAVCAAGEAKKLGVRPPDF

>LylinOBP9

DEQTNAMVAKAFNKCHGEFPIGDDEMKGVREKSTVPDSHNAKCLMACMLKEGKILRDGKYEKENAIVMADVLNKDDPAAADKAKQLVETCATQVGSDASADECEFAYKMAVCAAAWSSSTRFLKTHPFLLQLCTHRSTWHTFKYEYLPGNTSSRDLTHHINIVCVT

>LylinOBP10

MTPIVAILFALLAAHVKAYKKSGGIDANGKYSIEGFNKLVDMKYKGEENAGAKMIVKDCSSKVAPKEGEKCSVGRTIRECLSAASKENEFFTI

>LylinOBP11

APSANVKEIVQNVSKKCVAETKASPEQAKIAVSQHIPKDDVERCYLQCVYTGVGVIKDGKFSEEGGKKLVALRFHDAKEKELANKLIATCAKEIKAKDGEKCSLGRAVRECFVNHGKQVNFFPSA

>LylinOBP12

MVAECPAYSWVCQSTLSYYLHLQQTLQYIDRDPTLAMNQSSCILTLALTIFVMVVVSGFKELDSVLPQAKQEECRKESNFQGELSGDVSQNVTQELKCFAACSLVKLGLMNEKDGTINTTQLDELIAKHTAGKDAADMFKHSVVEPCLKEVNKTADYCEYSFQLVTCGMNKVKPPTTG

>LylinOBP13

ITPELDKKAKEAVAKCADVPGINEAKKEDCYAACFMTEMGYMTDGKINVENMEEANKQKWDDQQMINKGIEIDKTCAKQVGDTKGKSECAIGYDFGVCKTRLVKANCILRSILQTQLVPSPWWAHLPPLTPLKDSSTFNASFLKGVYREDMEQRNYNKTGLQPPTPLKQ

>LylinOBP14

ITPELDKKAKEAVAKCADVPEINEAKKEDCYAACFMTEMGYMTDGKINVENMEEANKQKWDDQQMINKGIEIDKTCAKQVGDTKGKSECAIGYDFGVCKTRLVKANCILRSILQXQLVPSPWWAHLPPLTPLKDSSTFNASFLKGVYRGDMEQRNYNKGKNKTGLQPPTPLKQ

>LylinOBP15

ITPELDKKAKEAVAKCADVPGINEAKKEDCYAACFMTEMGYMTDGKINVENMEEANRQKWDDQQMINKGIEIDKTCAKQVGDTKGKSECAIGYDFGVCKTRLVKATGLQPPTPLKQ

>LylinOBP16

ITDELRKKATEARLKCKQQVGLSDKEYQDWVKGISLPITNGGSCCEVCACWMRELGYMTDGHLNLNNMKNVNTQKWSEKANVEKANQIDTLCTARVVQDGRKECEIALDYRKCKTEMIKQNGGPPKPGST

>LylinOBP17

TDELRKKATETRLKCKQQVGLSDKEYQDWVKGISLPTTNGGSCCEVCACWMRELGYMTDGHLNLNNMKKRQDKYKHS

>LylinOBP18

MRSTGSECFEEVDAKLGNKTSWESDMDPYNCEKVKRMKKRHYCLHECKAKKLGVANEEGVLDFPKVKDLLLSRVNETWQKDILGQAADTCANSKFDQTWKDDTEEYKCNPQAIQFKHCVWKQVEMKCPEEHQNTGRHCKKLRSKISSETSKDSTAKETSV

>LylinOBP19

VTKEYHDRAVAAKDKCAKEHNIKESEIQEFVKKHKLPETEDGKCMIACYMEEMKLITDGKVNVDEWKKSNKEKWDEEAHVAMADEIVDKCNEQVSPDGLAKCEYGFKLTECGLKHRLEKGLPAPNMDDVKRR

>LylinOBP20

MKLVKDDKARPPKPEGYECIDDCIMAKNGFLGTDKKIDAAKVNAAAKTSYTGEWAEPGAKMVEKCLAQVSANKEKGECTSGADIFSICMFRESFINCPEKSWTSSETCKANKERLIKCPKSIPFLNKSAK

>LylinOBP21

QMDEDPDCRPPHPPGKEAQCCPLPDFVGVVDNFHDVMHKCSDEAGLRKPSGPPGSGTPPTAEEMAAHMSAHECADECLFKNTKYLQSNGELDKDAIKASVTKIFTGDWAALASSAADKCLASAKSEVGASAKCKSGARQMVKCFTRAMFLNCPASSWTESTECAAAKARITKCPNAMVPMIPPYPQPISANSTILDSLFACITDGEYMEFQSISSGRLG

>LylinOBP22

MPSQLPCPFFLLNIAFAHPGHFDEDPECRQPHHHRHEENDCCKVPSLFSNNKDEMHELVHKCFEEAGIKKHGPHHEHHGPPPLEDGPIPPPPPPPFSPKNDSKLDCVEQCFLKNLDLVDDEGDLKVDDLKALVTEKFSGDWASVGSSAIEKCLEKAKTEENESSKCKAGSKRVLHCHCTQHASYPKMGGSQSARKVTLDNPNPDPHDLNNVI

>LylinOBP23

HPGHFDEDPECRQPHHHRHEENDCCKVPSLFSNNKDEMHELVHKCFEEAGIKKHGPHHEHHGPPPLEDGPIPPPPPPPFSPKNDSKFDCVEQCFLKNLDLVDDEGDLKVDDLKALVTEKFSGDWASVGSSAIEKCLEKAKTEENEPSKCKAGSKRVLHCLAREFFMNCPASDWTESEVCLAAKDRVSKCPHSLPPMHH

>LylinOBP24

MADSVGECMKLIKVKPEKGPPVPEGFDCMDTCVFSKLGFIGADNKLDPEKLAKKFSELFKGDWSALSESTLKKCLPMADVGKGVCSSGADVFKFCLIRELYMNCPASSWTKSDLCKANVERLEKCPNSLPFMNGSGLKNKSSR

>LylinOBP25

QMDEDPECRPPHPPGKAGDCCVQPKLFDEGDMPDVIKKCHEEAGVKRPSGPPGSGTPPTAEEMAAHKSAHECAAECIFKNNNFIKSDGELDXDAIKATVTKMFTGDWATLASTTIDKCLASAKSEVDASPKCKSGADQVVRCFGRSLFIGCPASAWTESTECAAEKARLTKCPNAMPPPPHHKH

>LylinOBP26

FKELDSVLPQAKQEECRKESNFQGELSGDLSQNVTQELKCFAACSLVKLGLMNEKDGTINTTQLDELIAKHTEGKDAADMFKHSVVEPCLKEVNKTADYCEYSFQLVTCGMNKVKPPTTG

>LylinOBP27

KFYRRFKHHQIAAHLTFCVYVKTGSRDKTETPRSERPLCKAPTSAPRKLEKVINQCQEEIKYALLQEALSVLGETVSLRTALTRNRSKRETFTGEERRIAGCLLQCVYRKMKALDETGFPTATGLVKIYSEGVEDRNYYLATIQGVQQCLSRELQNRNKNPSIVKAEGYSCDVAYDVFNCVSEEIEQLCGTSP

>LylinOBP28

VDEKRPLCKAPTSAPRKLEKVINQCQEEIKYALLQEALSVLGETVSLRTALTRNRSKRETFTGEERRIAGCLLQCVYRKMKALDETGFPTATGLVKIYSEGVEDRNYYLATIQGVQQCLSRELQNRNKNPSIVKAEGYSCDVAYDVFNCVSEEIEQLCGTSP

>LylinOBP29

QQEDCKTAPAGWPKRPPQCCDLPFPLEGMKREFGSCIRQIGNRQSSAVPTAQAVRDARLCIEECVYKGLGFMEEHNLNKDQLLEQLKKGVAGKKDWEKPMEDAVKSCHETITKRETPQEGACQDSAHEFTHCVMRQLFLSCPASEWNNNDECNLVKNRMQACPNIPPPPPPPPQGFRGQGPPQPQ

>LylinOBP30

NPTTPNPTSSSHAASVSGGSTVSGVSKSPEEVKQKIKEQVEALTGACKSQTKITGEQAKIVATQAIPKTEAEKCFLECIYTGLQLTKDGKFNEPAARALAQKRFGNAPEDLTKANSMIDTCVKEVVVKDLNEKCALGRLIRECFVKNGAKINFFPKP

>LylinOBP31

QMDEDPDCRPPHPPGKEAQCCPLPDFVGVVDNFHDVMHKCSDEAGLRKPSGPPGSGTPPTAEEMAAHMSAHECADECLFKNTKYLQSNGELDKDAIKASVTKIFTGDWAALASSAADKCLASAKSEVGASAKCKSGARQMVKCFTRAMFLNCPASSWTESTECAAAKARITKCPNAMVPMIPPKH

>LylinOBP32

MEVNAERNVTDEQRXAVRLCSRYTEVESGLAEAGYDCLAECFFIKLGLMGEDKTLNKENILEEVRIQFHEDXVEPARKALETCMEKKYNTKCPSGIDGTMQCFTVQLMLNCPXQNWTDGEECKETRTFMEKCGETLNYYD

>LylinOBP33

LEKVINQCQEEIKYALLQEALSVLGETVSLRTALTRNRSKRETFTGEERRIAGFEFRCDLXDGWKWSQEERGGSSPTWFEINTIPGMNTTSRSWRLSSQISSSSVFCNVYTGK

>AfasOBP11

ISKEYHDKAVAARTTCAKLHNVDDKTIMEHWKNHQLPEKEPETCIIICYLKEMKLVVDGKVDADAWKASNKEKWDDEKHVAAADEIVDKCSAEVPPTENECEWGLALTKCGLKHGKEAGIPPPDMEHPKRR*

>AsutOBP12

MFQAFVYQKFLKETIKKCQKGKNVTDDEVEEFTKPLVPKNEKERCLVACLFKEYKVIIDGLFVPFNALNVAKVVYKDYPDKVERIKDVLDHCGEDIPSHNDNECDLAGDIMKCEVKYLNSIPKMTSLEFLAGSMAATAEP

>AsutOBP11

AYHDKAIAAKNTCAKLHNVDDETIMKFWKAHQLPEKEPETCIIICYLKEMKLVVDGKVDADAWKASNKEKWDDEKHVAAADEIVDKCSAEVPPTENECEWGLALTKCALKHGKEAGIPPPDMEHPKRR

>AsutOBP8

EGNINKEYLDKLIAAKEKCVKEFSVDDSIVEDLYVRYNKPPTESGKCMVACYMEERGMMKDGKTITEQVMLDNQEKWIAATHVNMGKEVIDTCDKEVPNEENDKCDLAVDYMMCLVKRGDEAGLPKMDVAQLKH

>AsutOBP6

KELTDEQKEQIFAEIKNCMESTKLTDEEFESIMAKKELPTSKEGKCFTKCLMEKMEYLEEGGKINVIAVQAGLEENMEKESEITKAKEIIQQCADTVPPEDSCEYAYGISQCMYTKMKEAGISGGP

>AsutOBP10

QELPPPGDVKNKTVVFKNSFLRSAKYCSSIYETSTLAIMALLMSEKSDDQNGKCFLNCMLQRYRLMSQDGSYNKDKFKPFLEYIPDSKFLQPIRGNLKNCISEKDPDPCEKASKFVKCFYTRARNKGEIGASKEVIPADG

>AsutOBP7

QQEDCKTAPAGWPRRPPQCCYLPFPLEGMKKEFGSCIRQIGNRQSSAVPTAQAVRDARLCIEECVYKGLGFMDEHKLNKDQLLEQLKKGIADKKDWTKPMEGAVKKCHETITKRETPQEAACQDSAHEFTHCVMRELFLNCPASEWSNNDECNLVKSRMQACPNIPPPPPPPPQGFRGQGPPPQ

>AlucOBP1

VINEECKDRNQSSTEYETFYNCCDLESSFNETKSKEKEEAREFCENEFEKANNVSEDEGPSPASVGQDCYFECVLKKIGAMSEDYKMDKEKVTKWFMEGSHKDFEEVGKQAMEKCYDKTYSKKYCASGVMGLLWCYSEEIVMNCPAKYWDQSEKCTAAKAYMKKCSTNPWRSED*

>AlucOBP2

MRSTGSECFEEIDAKLGNKTSLESDMDPYNCEKVKRMKKRHYCMHECKAKKLGVATEEGNLEFPKVKELLLSRVNETWQKDILGQAADTCATSKFDQTWKDDTEEYKCNPQALQFKHCVWKQVEMKCPEEHQNTGRHCKKLRSKISSETSKDIAKETSV*

>AlucOBP3

QPDEDPECRPPHPPGKDDKCCTIPELIVGENMQAMMKQCFEESGMERRPPGPPGPPGSGTPPTPEEIEAHRSAHECVDECFFKAAKFMNSDGEFDLEAMKTAAASVFTGDWAPLGSETIDECFASAKSQVSASAKCTSGAHQAKKCILRNFIINCPPSAWNDSTDCAALKARLTKCPNAMPPFPHHKH*

>AlucOBP4

VEEGRPLCKAPTTAPRKLEKVINQCQEEIKYALLQEALSVLGETVSLKTALTRNRSKRETFTGEERRIAGCLLQCVYRKMKALDETGFPTATGLVKIYSEGVEDRNYYLATIQGVQQCLSRELQSRNTNPSIVKAEGYSCDVAYDMFNCVSEQIEQLCGTSP*

>AlucOBP5

NPTTPNPSTSHVSSSAGITVSGVSKSPEEIKLKIKEQVATLTGACKTQTKLTGEQAKIVASQAIPKTEAEKCFLECIYQGLQLTKDGKFNEPAARALAQKRFGNAPEDLQKANTMIDICVKEVVVKDENEKCALGRLIRECFVKNGAKINFFPKP*

>AlucOBP6

APPEEPAECKIPESDSAELVKCCKLNVVLDEMADSVGECMKLVKGKPEKGPPVPEGFDCMDTCVFSKLGFVANNKLDAEKLTKKFSELFKGDWSALSDSTLKKCLPMAEGAKGSCASGADVFKFCIVRELYMNCPASSWTKSDLCKANVERLEKCPHSMPFLPGTGIKKN*

>AlucOBP7

EEQANALVAKAFNKCFGEFPLGDDEMKEVKDKSTVPSSHNAKCLMACMLKEGRILRGGKYELENAILMADVLNKNDHAAADKAKQLIETCAAQVGTDASADECEFAYKMALCASDEAKKLGVRPPDF*

>AlucOBP8

VMTQAQMKQAMKTVRNMCIPKSGVDKEALAKMVEGEFDESDQKLKCYLGCVLGMMQAVKNNKINLTMVKNQISKMLAPEQGQRILAAFEGCATVTGDDNCDLAFKFAKCIYDTDKELLFQAFIVP*

>AlucOBP9

ITKEYHDRAVAAKDACLKKHPSIKESDVQEFLKKHKLPETDDGKCMIACYMEEMNLMADGKINVEEAKKTNSDKYDGEPDNKELADKLIDHCSSQVSPDGMSKCEYAYQFSKCGLEYGMKNGLTPPKMYEEQRR*

>AlucOBP10

MTYHVFFRKFDLPRISRRVRQCYYHSVPRSLSGSSRRMLEETSQHHPKRRSRVSEKHKLPETDDGECMIACYMEEKNLMADGKINVKEANQTNSDKYDGEPDNKQLAEKLIDHCSSQVSPDGMSKCEYAYQFSKCGLEYGMKNGLTPPKMYEEQRR*

>AlucOBP11

APTDDMAACMQITNEDSASMATCCDYVIPFSNKTMTTCDKKETSGEMSKEFECVQDCLFSSDNVLGADKKFDPVAWRKHATNTISGDWKEVIANSGSNCEGFKKVLAQSMEKKCPTSESDVSFNCMTLQWYMNCPKSAWTSSESCEASKKKLMSCFGPIFENTS*

>AlucOBP12

EINEECKDIENLKTQLENFYGCCDFESMIERVVRTAEEVETDRFCREERKKINSTDGKMPLASEGHDCFMECVLKRMGAMGQDFKFIREKLDDFFLRGYPEEVKQAGKLAFDKCLSKNFSKKYCASGINGLMMCLPEELVMNCPANIWSSHESCPIAKEAIKKCPSYRVMIEQE*

>AlucOBP13

FKELDDVLPKPKQDECRKESNFQAELPSDINQNITQELKCFAACSLVKLGLMNEKDGTINMAQLEDLIAKHTGGKDAADMFKHTVVEPCMKEVNKTTDYCEYSFQLVKCGMSKVKPPSTGTEG

>AlucOBP14

YQEVLKATLKDCKGGKEITQEEVDEFMKPLIPKNEEERCLMACVFRAYNVIVDGHFDPKLAYGVAKNILHENPEKLKHIKETLDYCGHEIPTKMDNECDLAGEVMSCRNKYNIDHGYDQDP

>AlucOBP15

ITPELDKRAKAAVAKCADVPRTDEAKKEDCHAGCFMSAMGYMTNGEINVKNMEEANKQKWDDQEIIKKGIQVDTTCAKQVGDTKGKSECTIGYEFSTCKKELVKKVGLPPPTPLKE

>AlucOBP16

ITDELKQKAQAARLTCKQQVGLSDKEFNDWVKGIALPTTDGGTCCEVCACWMRELGYLTGGRVNLENMKAVNAQKWNNLAYVELGNKIDALCSDRVLQTGRKECEIAVDFRKCKTELIQQFGGPPKPGST

>AlucOBP17

GELPEEMREMAQGLHDSCVGETGVDNGLIAPCAKGSFADDPKLKCYFKCVFGNLGVISDDGELDAEAFASILPDNMQALLPTIRGCGSTTGADPCDLAMNFNKCLQKADPVNFMVI

>AlucOBP18

APSANVKEIVQNVSKKCAAETKASPDQAKIVLSKNIPKDDAERCFLQCVYTGVGVIKDGKFSEEGGKKLVALRFHDAKEKELANKLIATCAKEIKAKDGEKCSLGRAVRECFVNHGKQVNFFPSA

>AlucOBP19

GNIKEGYVAKIAEIKDKCLKEHNVDHSVVEDLLKKSIKPEVKAAQCMVACFFEENGMMKDGKIVSEMVKSNNAHQYEDPADVEKANEASDMCDGEVSTDGKDKCLLAADYALCWVKRTEEAGLPQIDFANSS

>AlucOBP20

APPADEPAECKPMKEKEEEISKCCKLEPVTVKEQAAFVDCMKLVKDTDKKGPPKPEGFECLDDCILSKTGSLGSDKKIDPAKINAAAKTTYTGDWAEPGAKMVEKCLAQVAENKDKTVCSTSGADVYTKCIFRESYINCPEKSWTNSDACKANKERVIKCPKTLPYNAEQHKAETR

>AlucOBP21

NEKKANEKVTEIFNKCKETWPVTDEEIEQVKQKQSIPDSKNVKCILACMLKEAKILRDGEYNKDNAELMADVLYKDEPEHAEKSKQIIEMCSSELGTKTEGDDCEYAYKMSVCASKHAKELGVKTPEF

>AlucOBP22

YQDQLKQTIKDCQGGKEVTDEELEEFTKPLIPKNEEERCIMACVMRTYNIINNGHYDPKIAFGIIKGILKDHPEKLDRIKEVMDHCGEDVPQHMDNECDLAGEIMQCEVKYQKAMGLN

>AlucOBP23

HPGHFDEDPECRPPHSHRHEEKECCKTPNLFSKNKDEMHELVHKCFEEAGIKKPHHGHHGPPPPGDEPPPPPPPPFHSKNNTKFECVEQCFLKNLELIDEEGDLKIDDFKALVGEKYTGDWASVGSAALEKCLEKTKTEEKESSKCKAGSKHVLLCIARESFINCPASDWTESEVCSDAKERVVKCPDIPPPMNH

>AlucOBP24

YQEQLKETIKQCQDGREVTDDEVEEFTKPLVPKNQEERCLVACVFKEYKVIIDGHFDPVNALNVAKMVYKDYPEKWKRIKDVIDHCGEDIPTHNDNECDLAGDIMNCEVKYLNSMPKGVSLELLAGSIAATAEP

>AlucOBP25

QMDDDPECRPPPPPNKEGSCCTVPRLLDNADKPEVIKKCHDEAGMKRPSGPPGSGTPPTAEEMAAHKSAHECADECIFKSSNLLKSDGELDQDAIKATTTKMFTGDWSTIASTAVEKCLATAKSEVGASAKCKSGAHQMVKCFARTMFLNCPASSWTESTECAAAKTRLTKCPNAMPPPPHHSRH

>AlucOBP26

NTKELSPSEALKQKVKVQCQQEVKATPEQLKIYDNFKDVPKDDVENCLMECMYTKTGGIGADGKYSVEGFKKLVDMKYKGEENTKARKIAADCEAKAAPKEGEKCSMGRAIRECLAAATKENEFFTI

>AlucOBP27

KELTEEQRTQLFEDLKQCKNSTDLSDDEFETIIAKKELPTSEAGKCFTKCLMEKLDIIEDAEGGKKKISVITMQASLEENMEKEDDIAKGKDIIQKCGDTVEPEDSCAYAYNISKCIYDRMKEAGISQ

>AlucOBP28

MIIEIICVLTVGISPHFIEGQELPPPGGVGNKTAVFKESFIRTAKYCSSIHETSTVAVLAILMSEESDDQNGKCFLNCMLQRYQLMSKQGAYNKDKFKPFLDYIPESRFLQSIKGNLKTCITERDPAPCEKAYKFIKCFYTRARNKDEFGKIQRK

>AlucOBP29

QQEDCKTAPAGWPRRPPQCCDLPFPLEGMKKEFGSCIRQIGNRQSSAVPTAQAVRDARLCIEECVYKGLGFMEEHNLNKDQILQQLTKGVADKKDWTKPMEDAVKSCHETITKRETPQEGTCKDSAHEFTHCVMRQLFLSCPASEWNNNDECNLVKSRMQACPNIPPPPPPPPQGFRGQGPPPPQ

>AlucOBP30

TPTTPTPATSRRVTVAPEDLEQAKSLRKFCTAKTGFTGITTTETSKGKDQARTATTPRPKTQLEKCYLECLYTGLQLTKDGKFNEPGARALANKRYKNAPEELRKVNSIIDFCITEVVVRDIEEMCALGRLIKECFSKYGAKNFPEL

>AlucOBP31

QIDEDPECRPSGPPGKEPECCTIPMKLFGDEVQEAVVKNCFDEAGMKRPSGPHGGGSPPTAEEMAAHISAHECADECVFKSGNFIKSDGGLDEDAIKAVIAKLFTGDWAPIATAAVNKCLASAKSGVSASAKCKSGAYQLSKCFQRELFLGCPASLWTESTDCSAIKARITKCPNAKVPIGHHHKH

>AlucOBP32

EVLTDGDCPKTMPKEMKPLYKCCVVEMDSNKTISDDQKAAVDSCVNTSKSDSDANKHDCMIECIFIKLGYMGEDKTINVDYVLKEMNSLLPEDFHEQTSKSLATCMGKKFSSTECPSEIDGVMACFSTMVLMNCPAKHWTDDEECKATRKFFQKCGDSIGYRYD

>AlucOBP33

GLPFQNEMAVMQCKVKFDVTAEDIQLLKDSKLPSSHSGKCMMACILKKMKVMTKRGQFDLRNVQKWLRNKYQGDQANLAKGNYVAEACANTLPTLGIQDECEMAAEIMTCVRTKSKLVKKTLNGELPKEVSP

>AlucOBP34

AQKSKQPSKAKTKESQVVAARPKDARAAACVTQIGPDEEEEASFYRKEIPETDKGKCLLACYLESKGVLSGGKFSSSGAAKIAARAYPNNAAKTGNVKHILSHCGTIAARETEQCQLAYRLAECTTTLADKFKL

>AlucOBP35

GFMEALIECKQQHHVSKEEAMTGESEEVKCFSECVLKKSGMMSDNNEFDEEKIQAEGARMIKNDEQKNREFEGAAKACIEKVNGENPSEKCAKGHALFKCMKEAMPMSKMRG

>AlucOBP36

VSKEYHDKAIAAKNTCAKLHNVDDETIMKFWKAHQLPEKEPETCIIICYMKEMKLVVDGKVDADAWKASNKEKWDDEKHVAAADEIVDKCSAEVPPTENECEWGLALTKCALKHGKEAGIPPPDMEHPKRR

>AlucOBP37

VINKDYLEKVVTAKDKCLKEFNVDDSVVEDFIVRYNKPQSESGKCMVACYMEERGMMKDGKTITEQVMLDNQEKWIAATHVNMGKEVIDTCDKEVPNEKNDKCDLAVDYMMCLVKRGDEAGLPKMDVAQLKH

>AlucOBP38

KELTDEQKEQIFAEIKNCMESTKLTDEEFESIMAKKELPTSKEGKCFTKCLMEKMEYLEEGGKINVIAVQAGLEENMEKESEITKAKEIIQQCADTVPPEDSCEYAYGISQCMYTKMKEAGISGGP
